# One Billion hiPSC-Cardiomyocytes: Upscaling Engineered Cardiac Tissues to Create High Cell Density Therapies for Clinical Translation in Heart Regeneration

**DOI:** 10.3390/bioengineering10050587

**Published:** 2023-05-13

**Authors:** Kiera D. Dwyer, Rajeev J. Kant, Arvin H. Soepriatna, Stephanie M. Roser, Mark C. Daley, Sharif A. Sabe, Cynthia M. Xu, Bum-Rak Choi, Frank W. Sellke, Kareen L. K. Coulombe

**Affiliations:** 1 School of Engineering, Brown University Center for Biomedical Engineering, Providence, RI 02912, USA; kiera_dwyer@brown.edu (K.D.D.);; 2 Cardiovascular Research Center, Cardiovascular Institute, Rhode Island Hospital, Alpert Medical School of Brown University, Providence, RI 02903, USA; 3 Division of Cardiothoracic Surgery, Rhode Island Hospital, Alpert Medical School of Brown University, Providence, RI 02903, USA

**Keywords:** hiPSC-cardiomyocytes, engineered cardiac tissue, tissue engineering, scale up, biomanufacturing, cell-dense tissue, myocardial infarction, cell/tissue transplantation, engraftment, heart regeneration

## Abstract

Despite the overwhelming use of cellularized therapeutics in cardiac regenerative engineering, approaches to biomanufacture engineered cardiac tissues (ECTs) at clinical scale remain limited. This study aims to evaluate the impact of critical biomanufacturing decisions—namely cell dose, hydrogel composition, and size-on ECT formation and function—through the lens of clinical translation. ECTs were fabricated by mixing human induced pluripotent stem-cell-derived cardiomyocytes (hiPSC-CMs) and human cardiac fibroblasts into a collagen hydrogel to engineer meso-(3 × 9 mm), macro- (8 × 12 mm), and mega-ECTs (65 × 75 mm). Meso-ECTs exhibited a hiPSC-CM dose-dependent response in structure and mechanics, with high-density ECTs displaying reduced elastic modulus, collagen organization, prestrain development, and active stress generation. Scaling up, cell-dense macro-ECTs were able to follow point stimulation pacing without arrhythmogenesis. Finally, we successfully fabricated a mega-ECT at clinical scale containing 1 billion hiPSC-CMs for implantation in a swine model of chronic myocardial ischemia to demonstrate the technical feasibility of biomanufacturing, surgical implantation, and engraftment. Through this iterative process, we define the impact of manufacturing variables on ECT formation and function as well as identify challenges that must still be overcome to successfully accelerate ECT clinical translation.

## 1. Introduction

It is estimated that a massive 1 billion cardiomyocytes are lost following myocardial infarction (MI) [1]. What makes MI even more deadly, however, is the lack of native heart regeneration, as there is only ~1% turnover of cardiomyocytes per year in the young adult mammalian heart, which further declines with age [2]. Although there is evidence that cardiomyocyte proliferation increases slightly after MI, such limited regenerative capability does little to recover the cells lost during MI and thus contractile function [3]. Clinically, patients are increasingly surviving acute MI episodes due to quicker diagnosis and improved emergency interventions [4,5,6]. Because of the permanent muscle loss caused by ischemic injury, however, post-MI patients are at increased risk of heart failure (HF) development (20–30% diagnosed at 1 year [7,8,9]) and severity (4-fold increased risk of cardiovascular death [7]), with myocardial loss from MI as a main predictor of both (5% increase in infarct size associated with 20% increase in hospitalization for HF and 19% increased mortality [10]). With ~50,000 HF patients in the United States becoming refractory to medical treatment each year, there is a critical need to develop novel therapies to restore contractility and enhance cardiac output after ischemic injury [11]. 

Regenerative cell-based therapies are promising alternatives to traditional treatments. These cell therapies introduce embryonic or pluripotent stem cell-derived cardiomyocytes (PSC-CMs) into the damaged region of the heart to remuscularize the area, recover cardiac function, and limit disease progression to HF. Although the goal and rationale of cell-based cardiac therapies are conceptually simple and have been pursued for over a decade in preclinical studies, their implementation in the diseased heart remains incredibly challenging and nuanced. A major reason for this dichotomy is the fact that what makes the post-infarcted heart a great candidate for stem cell-based regenerative therapies also makes its application challenging. For example, the lack of intrinsic cardiac regeneration necessitates extrinsic therapies to regenerate the cellular composition, vascular bed, and/or structural microenvironment of the heart. Further, the massive loss of cells during MI lends itself to stem cell applications, as advances in the direct differentiation of PSCs into CMs to achieve high purity and numbers provide a virtually limitless source of starting material. Yet, moving towards clinical implementation requires advanced biomanufacturing and tissue engineering approaches to handle the large number of PSC-CMs. The most fundamental of these hurdles is consideration of the cell dose that can be delivered by direct intramuscular injection or epicardially within engineered cardiac tissue (ECT), as CM dose has a great potential impact on cardiac function. Because injected PSC-CMs produce arrhythmias, including ventricular tachycardia, while ECTs develop an electrical syncytium and are structurally whole upon implantation, ECTs have a lower arrhythmia risk, and their preclinical development for translation to patients is well justified.

Recent work in the field supports the notion that PSC-CM treatment has a therapeutic window, or dose of cells, that improves cardiac function after injury. Querdel et al. were one of the first to investigate the impact of CM dosage on cardiac function in vivo [12]. Using a cryoinjury guinea pig model of MI, Querdel et al. implanted ECTs with a low (4.5 M), medium (8.5 M), or high (12 M) hiPSC-CM dose in vivo, finding a dose-dependent effect on remuscularization of the scar (low: 1.4 ± 0.4%, medium: 5.3 ± 2.0%, high: 12.3 ± 2.5%) as well as functional improvement assessed by left ventricular (LV) fractional area shortening (FAS), with only the highest dose having a significant impact (+8% absolute, +24% relative increase). Upscaling these results to patients, it is estimated that ~1000 × 10^6^ (1 billion) CMs are required to have a therapeutic impact on the post-MI adult human heart [12,13]. Despite the importance of scaling up ECTs in terms of cell dose to maximize therapeutic impact as well as size for clinical relevance, the design parameters have not been adequately defined for manufacturing cell-dense ECTs that can accommodate the 1 billion CMs lost during MI in patients. 

To date, the limited number of rigorous studies exploring ECT scale-up poses an immense hurdle to maximizing the therapeutic potential of this technology. Simply assuming that scaling up is linear oversimplifies ECT biomanufacturing, as multiple interconnected factors such as cell density, size, extracellular matrix concentration, and time impact tissue formation, function, and potential treatment efficacy. Thus, the design space for fabricating ECTs of clinically relevant cell dose and size must be defined, and novel solutions must be developed for the gaps that emerge upon scaling up. In this study, we calculate cell dose per volume and use hiPSC-CM density as the independent variable. With high-throughput small-scale ECTs (meso-ECTs), we report the significant impact of hiPSC-CM density on tissue formation and function. Scaling up to a larger, planar surface area (macro-ECTs), we additionally evaluate the impact of hiPSC-CM density on electrical signal propagation and tissue arrhythmogenesis risk. Lastly, we demonstrate the technical feasibility of engineering and implanting a single-entity mega-ECT composed of 1 billion hiPSC-CMs in a swine model of chronic myocardial ischemia. Our work highlights key criteria for the fabrication of ECT therapies at a clinical scale and provides a foundation for further translational studies to engineer cell therapies that maximize the potential for functional contributions of ECTs to restore cardiac function after injury.

## 2. Materials and Methods

### 2.1. HiPSC Maintenance 

The WTC-11 GCaMP hiPSC line (Bruce Conklin, Gladstone Institute, UCSF) was utilized for all experimental work. For maintenance culture, WTC-11s were seeded in 10 cm^2^ dishes coated with 5 µg/mL vitronectin (Thermo Fisher, Waltham, MA, USA) and maintained in Essential 8 (E8) medium (Gibco) within a cell culture incubator (37 °C, 5% CO_2_). When WTC-11s reached 80% confluency (4–5 days), the cells were passaged using Versene (0.5 M EDTA, 1.1 mM D-glucose; MilliporeSigma, Cleveland, OH, USA) in Dulbecco’s phosphate-buffered saline (DPBS; Gibco) without calcium and magnesium. 

### 2.2. HiPSC-CM Differentiation, Freezing/Thawing, Expansion and Selection 

HiPSCs were differentiated into cardiomyocytes using the well-adopted, chemically defined small molecule protocol [14,15,16,17]. Briefly, upon passage, hiPSCs were replated on 0.1 mg/mL Geltrex-coated (Gibco) 24-well plates in E8 medium with 10 µM Y-27632 (Rock Inhibitor, RI; Tocris, Bristol, UK). The next day, hiPSCs were treated with the Wnt activator CHIR 99021 (3–5 µM Chiron; Tocris, Bristol, UK) in CDM3 media [15] for 24 h. Then, the Wnt inhibitor IWP2 (5 µM, Tocris, Bristol, UK) was added 72 h after Chiron treatment. The differentiating cells were fed with CDM3 every other day until day 9 when the media was changed to RPMI/B27 (Gibco).

At day 11, hiPSC-CMs were harvested and prepared for cryogenic storage to generate cell banks. For harvest, hiPSC-CMs culture plates were washed with DPBS followed by incubation in TrypLE™ Select Enzyme (TrypLE10x; Gibco). Cells were lightly triturated before transferring to a collection tube with an equal volume of RPMI/B27 supplemented with 10% Fetal Bovine Serum (FBS; Gibco) and 100 U/mL DNase I (DNase; Thermo Fisher Scientific, Waltham, MA, USA). Cells were counted with a hemocytometer and centrifuged (5 min, 300× *g*). The cell pellet was resuspended with CryoStor^®^CS10 (Stem Cell, Vancouver, Canada) at a concentration of 5–10 M/mL. Vials were placed inside a room-temperature CoolCell and frozen at −80 °C overnight. For long-term storage, cell vials were transferred to liquid nitrogen the following day.

For expansion, hiPSC-CMs from liquid nitrogen storage were thawed in a 37 °C water bath. The thawed cell solution was transferred directly to a large volume of RPMI/B27 and centrifuged (5 min, 300× *g*). The cell pellet was isolated and resuspended in RPMI/B27 + 5 µM RI. Cells were seeded on a Geltrex-coated plate at low density (~300,000 hiPSC-CMs/cm^2^ plate) in either 15 cm plates or T225 flasks. Proliferation of terminally differentiated hiPSC-CMs was induced using low-concentration Wnt activation (2 µM Chiron), which increased cell counts by approximately 3-fold [17]. Metabolic selection was then performed in glucose-free medium (DMEM (-) glucose) supplemented with 4 µM sodium lactate (MilliporeSigma, Cleveland, OH, USA) [18], followed by Wnt inhibition with 4 µM Wnt-C59 (Tocris). This protocol yielded cardiomyocyte purity >70% quantified by flow cytometry analysis of cardiac troponin T (cTnT) expression, with a range of 71.86–93.33% used in this study unless otherwise noted. All ECTs, including the mega-ECT containing 1 billion cardiomyocytes, were fabricated with hiPSC-CMs generated from this differentiation, expansion, and selection procedure. 

### 2.3. Cardiac Fibroblast Maintenance

Human primary ventricular cardiac fibroblasts (hCFs; Lonza) were maintained on 15 cm^2^ plates with hCF media composed of DMEM/F12 (Gibco), 10% FBS, 4 ng/mL bFGF (Stemgent, Beltsville, MD, USA), and 100 µg/mL penicillin-streptomycin (penstrep; MilliporeSigma, Cleveland, OH, USA). Upon confluency, hCFs were passaged using 0.05% trypsin (Gibco) in Versene and frozen back in hCF media with 10% dimethyl sulfoxide (DMSO; Fisher Scientific, Waltham, MA, USA). All experiments used 5% hCFs between passages 5–7, as informed by recent work [19]. HCFs were counted and viability assessed by trypan blue upon thawing before being used within ECTs. 

### 2.4. Molding System and Modification for Scale Up

**Figure 1:**
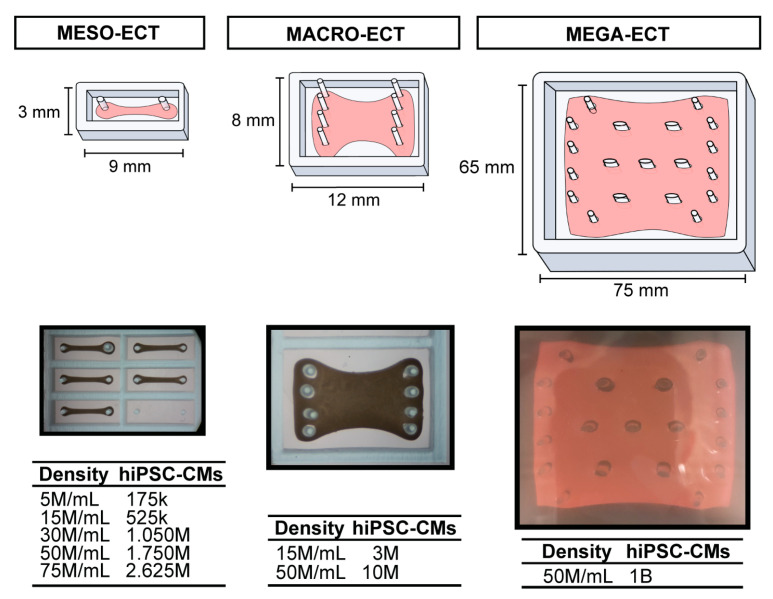
ECT scaleup. Meso-, macro-, and mega-ECTs with densities and cell quantities. k refers to thousand; M refers to million; and B refers to billion.

Polydimethylsiloxane (PDMS) molds for ECT formation and culture were created using a simple replica-molding workflow, as previously described [20]. Briefly, negative templates were laser-etched in ¼-inch acrylic sheets from vector-based designs created in Adobe Illustrator. For laser etching, a Universal Laser Systems 6.75 Laser Cutter (ULS) at the Brown Design Workshop (Brown University, Providence, RI, USA) was utilized to create acrylic templates. Sylgard 184 (PDMS; Dow, Midland, MI, USA) was then cast on the acrylic templates and cured overnight at 60 °C. The cured PDMS molds were removed from the acrylic templates and sterilized with an autoclave at 121 °C for 30 min. For this study, three molding systems were used for iterative, progressive scale up: (1) meso-molds: 3 × 9 mm, 35 µL; (2) macro-molds: 8 × 12 mm, 200 µL; (3) mega-molds: 65 × 75 mm, 20 mL (Figure 1). One hour immediately prior to casting, the PDMS molds were coated with 5% Pluronic^®^ F-127 (MilliporeSigma, Cleveland, OH, USA) in milliQ-H_2_O to facilitate homogenous tissue casting and mitigate wall adhesion.

### 2.5. Fabrication of Engineered Cardiac Tissue (ECTs)

To fabricate ECTs, hiPSC-CMs were harvested using TrypLE10x and combined with 5% hCFs from thawing. The cell solution was diluted in RPMI/B27 media to the desired density and combined 1:1 with a collagen hydrogel solution. Two collagen sources were used with different stock concentrations to enable increasing the collagen concentration (from 1 to 3.5 mg/mL) when scaling up from meso-ECTs (1 mg/mL collagen concentration unless otherwise noted) to macro-ECTs (3.5 mg/mL collagen concentration) in order to balance the increased cell densities and tissue size. A commercial stock of rat tail type I collagen (3.9–4.1 mg/mL; Advanced BioMatrix, Carlsbad, CA, USA) was utilized for the generation of all 1 mg/mL collagen hydrogel ECTs. The hydrogel casting mix was prepared by combining the collagen stock with 10x RPMI 1640 (1:10; Gibco), HEPES (1:100; MilliporeSigma, Cleveland, OH, USA), mH_2_O and finally neutralizing the mix with 5 M sodium hydroxide to a pH between 7 and 7.5. To achieve the collagen concentration of 3.5 mg/mL, however, a higher stock concentration of type 1 collagen was required. Therefore, collagen type 1 was isolated in-house from rat tails following a previously described protocol [21]. Briefly, rat tails were collected from Sprague-Dawley rats and stored at −80 °C for up to one year. For collagen isolation, tails were thawed at room temperature for 2 h. The exterior of the tails was washed with isopropyl alcohol, and collagenous fibers were harvested by twisting the tail to expose the tendon. Isolated tendons were washed in PBS and then transferred to 0.1 M acetic acid in mH_2_O at 4 °C with agitation (~120 rpm stir bar) for ~3 days to facilitate tendon degradation. After ~3 days, tissue remnants were removed from the solution through centrifugation (4700× *g* at 4 °C for 2 h). To the supernatant, 5 M sodium chloride (NaCl; MilliporeSigma, Cleveland, OH, USA) in water was added to a final concentration of 4% NaCl to promote collagen precipitation out of solution. After 1 h, the solution was centrifuged again (4700× *g* at 4 °C for 2 h), this time to isolate the pellet. The pellet was reconstituted at 26 mg/mL (concentration determined via lyophilization) in 0.1 M acetic acid. The collagen extracted from rat tails was sterilized by floating the solution on top of chloroform (10% *w*/*v*; MillporeSigma, Cleveland, OH, USA) in a bottle overnight at 4 °C. The collagen solution was then aseptically pipetted off the chloroform and stored at 4 °C for downstream applications. 

Once combined, the cell–hydrogel casting mix was immediately pipetted into the desired PDMS mold system and allowed to gel at 37 °C for 30–45 min. After which, the ECTs were cultured in a metabolic maturation medium (MM) as previously described [22], composed of DMEM without glucose (Gibco) supplemented with 3 mM glucose (MilliporeSigma, Cleveland, OH, USA), 10 mM L-lactate (MilliporeSigma, Cleveland, OH, USA), 5 mg/mL Vitamin B12 (MilliporeSigma, Cleveland, OH, USA), 0.82 mM biotin (MilliporeSigma, Cleveland, OH, USA), 5 mM creatine monohydrate (MilliporeSigma, Cleveland, OH, USA), 2 mM taurine (MilliporeSigma, Cleveland, OH, USA), 2 mM L-carnitine (MilliporeSigma, Cleveland, OH, USA), 0.5 mM ascorbic acid (MilliporeSigma, Cleveland, OH, USA), 1× NEAA (Gibco), 0.5% (*w*/*v*) Albumax (Thermo Fisher Scientific, Waltham, MA, USA), 1× B27, and 1% knockout serum replacement (KOSR; Gibco). ECTs were cultured at 37 °C, 5% CO_2_, and stimulated with a 4 ms biphasic field pulse stimulus at 1 Hz and 4 V/cm using a 6-well electrode insert (C-Dish, IonOptix, Westwood, MA, USA) connected to the IonOptix culture pacing system (C-Pace EP, IonOptix, Westwood, MA, USA) for up to 7 days prior to analysis. 

### 2.6. ECT Survival, Compaction and Pacing Analysis during Culture

Nondestructive, longitudinal brightfield optical microscopy images and videos (Olympus SZ40) were taken of ECTs daily. Survival curves were generated by monitoring tissue state throughout culture, with intact ECTs being defined as having no breakage either internally or at the PDMS posts. Survival curves and percent survival were calculated by plotting the number of intact tissues (as total minus broken tissues) over the 7 days of in vitro culture with Prism 8 (GraphPad). Using the daily images, ECT compaction was calculated by measuring the two-dimensional (2D) tissue area for each ECT in ImageJ, as previously described [23]. Two-dimensional areas were normalized to the area of the ECT on day 0 to calculate the fraction of 2D tissue area needed to assess the extent of compaction. Daily videos of each ECT beating in culture were used to count the number of contractile beats, which was divided by the time analyzed to calculate beats per minute (BPM). 

### 2.7. Mechanical Analysis

The passive (Young’s modulus) and active (contractile stress generation) mechanical properties of the meso-ECTs were analyzed using a custom micromechanical tensile apparatus (Aurora Scientific, Aurora, Canada). After 7 days in 3D culture, meso-ECTs were mounted on the tensile apparatus in a 37 °C bath of Tyrode’s solution. Tissues were stretched to 130% of their initial length in 5% increments and held for 120 s at each strain to allow for stress relaxation. During the last 20 s of this hold, an electrical stimulus of 1 Hz was administered to determine active stress contraction at 5% strain intervals up to 30%. At 30% stretch, the force–frequency response of the tissue was determined by increasing the electrical stimulus from 1 Hz to 4 Hz in 0.5 Hz intervals. A custom MATLAB (Mathworks, Natick, MA, USA) script was utilized to analyze active contractile amplitude (force, mN; active stress generation, mN/mm^2^) and kinetics (upstroke velocity, mN/mm^2^/s; time to 50% relaxation and 90% relaxation, ms). Contractile alternans were also quantified, defined as the change observed in contractile amplitude. The force–frequency response was recorded, with the maximum capture rate (MCR) as the maximum frequency at which the ECT could follow the target pacing. Meso-ECTs, as well as macro- and mega-ECTs that could not be securely mounted to the tensile testing apparatus, were analyzed using the video-based analysis software MUSCLEMOTION^®^ to determine contractile amplitude [24]. 

### 2.8. Curling Angle and Prestrain Measurement

Curling angle and prestrain calculations were made as previously described by Shi et al. [25]. After 7 days of in vitro culture, meso-ECTs being mechanically tested were removed from their PDMS posts, which anchor the tissue during culture. The meso-ECTs were transferred to a well plate with fresh media and allowed to sit at 37 °C for 10 min, defined as a “stress-free” environment in which the tissue could fully release any residual stress. Brightfield images were taken of the tissue to assess the different recoil profiles of density conditions. ImageJ (NIH, Bethesda, MD) was then used to quantify the curling angle of this tissue, which is defined as the angle made between the tissue endpoints and midpoints. To estimate prestrain in 1D, tissue displacement (u) was calculated by assuming the initial tissue position was fully linear (x, curling angle = 180°). ImageJ was then used to acquire x and y coordinates. From this displacement, the deformation tensor (1) and green strain (2) could be calculated.

F(x) = Δu(x)/Δx(1)

E(x) = 0.5 F(x)(2)

### 2.9. Immunohistochemical Staining, Imaging and Analysis of ECTs 

ECTs were fixed in 4% paraformaldehyde (MilliporeSigma, Cleveland, OH, USA) and washed with DPBS. For short-term storage, samples were kept at 4 °C. For staining, ECT samples were frozen and embedded in optical cutting temperature medium or processed in paraffin, followed by sectioning at 5 µm. Unless otherwise noted, ECTs were sectioned lengthwise through the full thickness of the tissue. Multiple sections throughout the ECT thickness (≥3) were used for staining. To visualize collagen content, picrosirius red/fast green (PRFG) staining was performed on sections, followed by imaging using an Olympus FV200 Slide Scanner. Following this imaging, second harmonic generation (SHG) imaging was performed on an Olympus FV1000 MPE Multiphoton Microscope to examine collagen organization. 

For immunohistochemical staining, sectioned samples were blocked with 1% normal goat serum (NGS; MilliporeSigma, Cleveland, OH, USA) in DPBS for 1 h followed by incubation with primary antibodies overnight at 4 °C. The following day, sections were incubated with secondary antibodies and nuclear counterstains for 1 h at room temperature. Primary and secondary antibodies are listed in Supplemental Table S1. Coverslips were mounted using Prolong AntiFade Glass Mountant (Invitrogen, Waltham, MA, USA). Once set, sections were imaged using an Olympus FV3000 confocal microscope and processed in ImageJ. Quantification of percent area was performed for all PRFG, SHG, and immunohistochemical targets. First, images were separated by channel, thresholded, and binarized. In the case of PRFG and SHG images, the percent area of the stain was normalized by the area of tissue analyzed, while sarcomeric α-actinin and wheat germ agglutinin (WGA) content was normalized by the number of nuclei. In conditions where sarcomeres were clearly visible, sarcomere length was determined by measuring the distance between 3–5 sarcomeres in a straight line and dividing by the number of sarcomeres. For quantification of content for the meso- and macro-ECTs, multiple (3–6) regions were averaged per ECT analyzed. For quantification of nuclei size in the meso-ECT and macro-ECTs, nuclei from multiple (3–6) regions were averaged (minimum 50 nuclei total) per ECT analyzed (represented by individual points on the graphs). For the mega-ECT, in which one tissue was analyzed, the quantification of content and nuclei is averaged per area analyzed (6 regions, represented by individual points on the graph).

### 2.10. Optical Mapping of Calcium and Voltage Transients 

To assess action potential (AP) and calcium kinetics in ECTs, a custom optical mapping system was utilized as previously described [19,26,27,28,29]. Briefly, ECTs are stained with the voltage-sensitive dye, di-4-ANEPPS (Invitrogen, Waltham, MA, USA) and imaged in a 37 °C temperature-controlled bath perfused with a solution containing 140 mM NaCl, 5.1 mM KCl, 1 MgCl_2_, 1 mM CaCl_2_, 0.33 mM NaH_2_PO_4_, 5 mM HEPES, and 7.5 mM glucose; gassed with 95% O_2_ and 5% CO_2_; and supplemented with 5 μM blebbistatin, an acto-myosin inhibitor to reduce motion artifacts. ECTs were paced by a concentric bipolar stimulation electrode that has a 1.3 mm diameter (small stimulation electrode set for mouse hearts, Harvard Apparatus). The stimulation strength was set to 0.3 mA for a duration of 2 ms. Fluorescent images of calcium tracing (using the intrinsic GCaMP signaling) and Aps were simultaneously recorded using dual CMOS cameras (100 × 100 pixels, 1000 frames/s, 10 × 10 mm FOV; Ultima-L, SciMedia, Costa Mesa, CA, USA). Calcium and voltage activation maps were generated from the maximum time delay between the cross-correlation of each pixel versus a reference pixel (±300 ms). The conduction velocities were calculated as the slopes of the line of best fit through distance versus activation time along the line. 

### 2.11. Animal Methods

**Figure 2:**
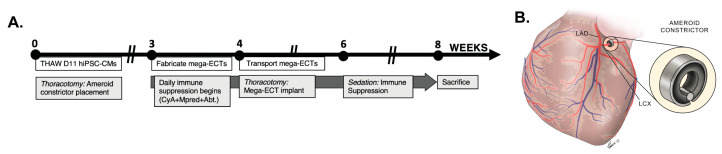
Preclinical swine model of chronic myocardial ischemia. (**A**) Timeline of in vivo swine study. (**B**) Schematic of ameroid constrictor placement on the proximal left circumflex (LCX) coronary artery to induce chronic myocardial ischemia in swine model.

All animal procedures were approved under IACUC #5024-21 at Rhode Island Hospital, according to the timeline in Figure 2A. Two Yorkshire swine (male and female; Tufts University, Medford, MA, USA), aged 8 to 10 weeks and weighing between 22 and 25 kg at the time of the initial procedure, were used. This pilot study was designed to assess technical feasibility using an established model of chronic myocardial ischemia developed by Dr. Sellke as previously described [30,31,32,33,34,35,36,37,38,39,40,41,42,43,44]. To induce chronic ischemia, a small left thoracotomy was performed, the pericardium was opened, and an ameroid constrictor composed of hygroscopic casein material cased in titanium (Research Instruments SW, Escondido, CA, USA) was sized and placed around the proximal left circumflex (LCX) coronary artery (Figure 2B). Slow swelling of the constrictor reduced blood flow, causing a slow onset of ischemia. Four weeks later, a second left thoracotomy was performed for implantation of the mega-ECT, which was secured to the epicardial surface with 4 sutures (6-0 prolene) at each corner of the mega-ECT. Heart rate and ECG activity were monitored daily throughout the study using a heart rate monitor belt (polar heart rate monitor chest strap). Our terminal endpoint was four weeks after the implantation surgery to assess the engraftment and impact of hiPSC-CMs. 

In order to promote engraftment and minimize xenograft rejection, we implemented an aggressive immune suppression protocol using cyclosporin A, methylprednisolone, and abatacept CTLA4 immunoglobulin, as successfully implemented by Laflamme and colleagues [45,46]. Trough measurements were taken throughout the study to monitor cyclosporine levels through a vascular access port (VAP) in the jugular vein that was implanted during the second surgery. 

### 2.12. Statistical Analysis

Student *t*-tests as well as one-way or two-way repeated-measures analysis of variance (ANOVA) were used in statistical testing as appropriate. When necessary, the Tukey–Kramer method of post hoc analysis was performed, with *p*-values < 0.05 considered statistically significant. All analysis was performed in Prism 8 (GraphPad) and reported with the standard error of the mean.

## 3. Results

### 3.1. Cell Density Impacts Meso-ECT Syncytium Formation, Structural Integrity and Collagen Remodeling

**Figure 3:**
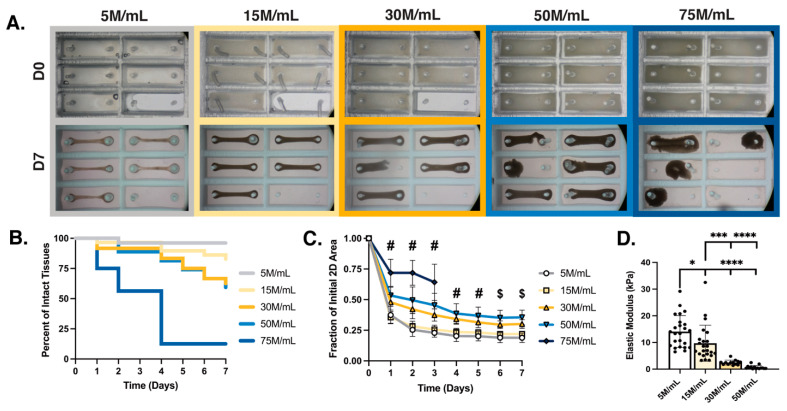
Increasing hiPSC-CM dose within meso-tissues decreases mechanical integrity and structural survival during culture. (**A**) Brightfield images illustrating tissue compaction from initial casting on day 0 (D0) to day 7 (D7) of in vitro culture (mold size, 3 × 9 mm); (**B**) survival curve showing percentage of intact meso-ECTs to assess structural survival; (**C**) quantification of tissue compaction over 7-day culture; (**D**) elastic modulus of tissues to assess passive mechanical properties with each point representing a single meso-ECT. 1 mg/mL collagen concentration utilized; M indicates million; *n* = 15–28 samples per group with significance defined as * *p* < 0.5; *** *p* < 0.001, **** *p* < 0.0001 with statistically significant comparison present between 5 M/mL and all other groups and 15 M/mL with all other groups; # indicates all conditions except the comparison between 5 M/mL and 15 M/mL are significant with a minimum *p* < 0.05; $ indicates all conditions are significant to each other at a minimum *p* < 0.05.

Motivated to develop ECTs with therapeutically relevant hiPSC-CM numbers, we studied tissue formation and function across a range of cell densities (5 M/mL, 15 M/mL, 30 M/mL, 50 M/mL, and 75 M/mL) using the standard collagen hydrogel concentration of 1 mg/mL within all our meso-ECTs, unless otherwise noted. From brightfield images of the meso-ECTs over the 1-week culture period (Figure 3A), it was evident that increasing cell density impacts tissue formation. Notably, increasing cell density decreased meso-ECT structural survival in culture (Figure 3B), with cell-dense meso-ECTs displaying greater breakage. At just 2 days in culture, structural survival of 75 M/mL meso-ECTs was only 56.25%, indicating that the initial stages of tissue formation and compaction are severely compromised with high hiPSC-CM input. After 7 days in culture, all but the highest density of 75 M/mL had >50% surviving intact tissues (5 M/mL: 96.15%; 15 M/mL: 82.76%; 30 M/mL: 62.50%; 50 M/mL: 59.26%; 75 M/mL: 12.50%). In culture, cell-dense tissues (50 M/mL and 75 M/mL) broke primarily due to the appearance of small tears in the meso-ECT interior. In contrast, hypercompaction and necking was the predominant failure mode in the lower cell density conditions (5 M/mL, 15 M/mL, and 30 M/mL). 

Over the 1-week culture period, the meso-ECTs compacted to varying degrees, a process in which the embedded cells form adhesions to one another and the protein matrix, generate intracellular and tissue-level tension, as well as produce and remodel the surrounding matrix. Increased compaction was observed throughout the culture in the 5 M/mL and 15 M/mL conditions compared to 30 M/mL, 50 M/mL, and 75 M/mL at every timepoint (Figure 3C). For all conditions, the majority of meso-ECT compaction occurred during the first 24 h of tissue formation. By day 4, compaction stabilized, as defined by no changes >5% (5 M/mL: 0.20 ± 0.01, 15 M/mL: 0.24 ± 0.01, 30 M/mL: 0.32 ± 0.01, 50 M/mL: 0.40 ± 0.02, fractional area). Comparison of input cell density with calculated cell density after compaction (day 7) was also quantified (Supplemental Table S2). Due to the particularly poor compaction and structural failure of 75 M/mL meso-ECTs, this group was excluded from further analysis. 

The structural integrity of meso-ECTs was next quantified by analyzing the elastic modulus, which showed an inverse relationship to cell density (5 M/mL: 14.09 ± 1.22 kPa; 15 M/mL: 9.71 ± 1.41 kPa; 30 M/mL: 2.46 ± 0.25 kPa, 50 M/mL: 0.67 ± 0.17 kPa) (Figure 3D). These values are lower than those of the native myocardium (30.80 ± 2.71 kPa in the longitudinal direction and 16.58 ± 1.85 kPa in the transverse direction), yet greater than the acellular 1 mg/mL collagen hydrogel controls (0.28 ± 0.03 kPa, mechanically tested after 24 h), suggesting increased stiffness with the addition of cells to the hydrogel, regardless of density. This inverse relationship between elastic modulus and cell density suggests that the low stiffness of the cell-dense meso-ECTs contributes to breakage, likely due to an imbalance of the cell:ECM ratio and physical space constraints (namely, cellular crowding) compromising adhesion formation and compaction that characterize tissue formation in vitro.

**Figure 4:**
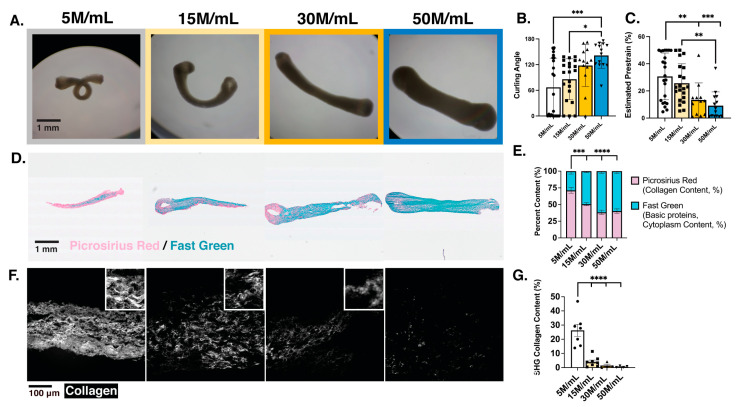
Structural integrity observed in different meso-ECT dose conditions is correlated with developed prestrain at the tissue level as well as extent and organization of collagen remodeling during tissue formation at the cellular level. (**A**) Brightfield images of ECTs showing curling in stress-free environment (removed from posts) after 7 days of culture; (**B**) quantification of ECT curling angle (degrees) and (**C**) developed prestrain (%); (**D**) representative histological staining of PRFG for each density condition; (**E**) quantification of the picrosirius red (collagen content) and fast green (basic proteins, cytoplasm content) per condition measured by % of area analyzed; (**F**) representative images of SHG imaging showing organized collagen fibrils; (**G**) quantification of collagen content imaged through SHG for each condition, measured by % area analyzed. 1 mg/mL collagen concentration utilized; M indicates million; *n* = 7–10 analyzed tissues per group for PRFG staining; *n* = 12–23 tissues analyzed for curling angle and prestrain quantification per condition; *n* = 4–7 for SHG with multiple areas averaged to analyze per tissue; the individual points represent single meso-ECTs analyzed; * *p* < 0.5; ** *p* < 0.01; *** *p* < 0.001, **** *p* < 0.0001.

Although reduced elastic modulus could be partially explained by decreased compaction of the cell-dense meso-ECTs, we sought to better understand the differences in the remodeled tissue environment, which may also contribute to these mechanics. At the global tissue level, we observed a different recoil profile of the meso-ECTs in a stress-free environment (Figure 4A). We measured decreased meso-ECT curling angles at higher cell densities (5 M/mL: 67.01 ± 14.19°; 15 M/mL: 85.95 ± 10.44°; 30 M/mL: 118.0 ± 13.59°; 50 M/mL: 141.6 ± 7.99°) (Figure 4B). As a corollary, prestrain, calculated from the tissue displacement in the stress-free environment, also showed that the highest cell densities had the lowest developed prestrain (5 M/mL: 30.68 ± 3.60%; 15 M/mL: 25.67 ± 3.12%; 30 M/mL: 13.32 ± 3.63%; 50 M/mL: 9.13 ± 2.71%) (Figure 4C). 

We additionally looked at the cellular scale by quantifying PRFG staining of the meso-ECTs (Figure 4D). The intensity of fast green stain (basic protein content, e.g., of the cytoplasm) showed an expected positive correlation with input density (5 M/mL: 28.80 ± 4.32%; 15 M/mL: 49.45 ± 2.29%; 30 M/mL: 61.59 ± 3.15%; 50 M/mL: 59.77 ± 3.33%, % area analyzed) (Figure 4E). Quantification of picrosirius red (collagen content), on the other hand, showed a negative correlation with input density, as the highest collagen content appeared in the lowest density condition (5 M/mL: 71.20 ± 4.32%; 15 M/mL: 50.55 ± 2.29%; 30 M/mL: 38.41 + 3.15%; 50 M/mL: 40.23 ± 3.33%, % area analyzed). Recognizing that not only the amount of collagen present but also its organization may be important in its contribution to the structural and mechanical integrity of the tissues, we quantified collagen organization using SHG imaging, which is an imaging modality that shows fibrillar collagen (Figure 4F). Collagen present in the meso-ECTs with lower cell densities (5 M/mL and 15 M/mL) was highly organized, as demonstrated by the bright signal. Similar to the PRFG results, the results of collagen quantification from SHG revealed the highest amount of organized collagen in the lowest cell density conditions (5 M/mL: 26.27 ± 4.26%; 15 M/mL: 4.08 + 1.24%; 30 M/mL: 1.49 ± 0.93%; 50 M/mL: 0.59 ± 0.27%, % area analyzed) (Figure 4G). No collagen abnormalities characteristic of fibrosis were observed in any condition [47]. An average ratio of the organized collagen content quantified by SHG normalized to the total general collagen content quantified through PRFG staining for each condition was calculated (5 M/mL: 36.89; 15 M/mL: 8.06; 30 M/mL: 3.88; 50 M/mL: 1.47, measured as SHG normalized to PRFG collagen content), illustrating a negative correlation between the organized-to-unorganized collagen ratio and increasing cell density. These results suggest that the organization of the ECM within the ECT is essential for the robust structural integrity of the tissue. 

To confirm that the observed changes in collagen content and organization were due to active cell remodeling as opposed to the persistence or development of the initial collagen hydrogel, PRFG and SHG imaging were performed on acellular collagen constructs. PRFG staining of the acellular constructs illustrated dissimilar collagen content and structure, supporting the hypothesis that active cell remodeling of the hydrogel was occurring within the low-density meso-ECTs (Supplemental Figure S1). Although SHG imaging was performed, no signal was obtained, suggesting the building of fibrillar collagen in the ECM occurs in ECTs during culture by the embedded cells as opposed to cell-initiated degradation. Additionally, because collagen deposition and organization can be influenced by the fibroblast-like non-myocytes arising from hiPSC-CM differentiation, we normalized our PRFG and SHG quantification by differentiation purity by multiplying the differentiation purity (cTnT+ as assessed by flow cytometry) and the quantification (Supplemental Figure S2). Through this normalization, the raw quantification values slightly decreased, with greater convergence between cardiac differentiation batches observed and statistical significance maintained.

Together, these results show that increased hiPSC-CM density in meso-ECTs negatively affects the ability of the embedded cells to remodel, compact, and develop prestrain within their environment, which compromises the passive mechanical properties of the tissue and increases its risk for structural failure. While there exists a threshold required for tissue integrity (to avoid structural failure), a spectrum of tissue compositions with varying stiffnesses and fibrillar collagen content is likely to be acceptable for implementation as a regenerative therapy. 

### 3.2. Physiological Analysis Reveals Cell-Dose Dependent Effects on Meso-ECT Contractile Stress and Sarcomere Structure 

**Figure 5:**
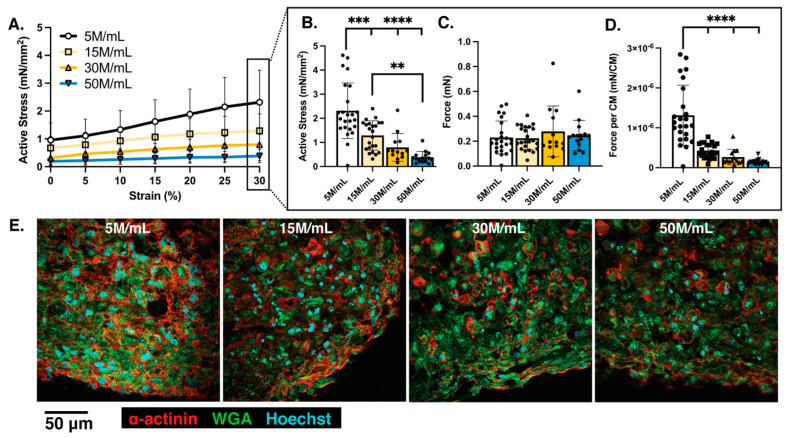
Increasing hiPSC-CM density within meso-ECTs alters normalized force generation, with histomorphological analysis confirming reduced sarcomere organization. (**A**) Active stress generation of ECTs from 0–30% stretch, measured at increments of 5%; (**B**) active stress, (**C**) force, and (**D**) force normalized by the number of hiPSC-CM within the ECTs at 30% stretch; (**E**) histological staining of α-sarcomeric actinin (α-actinin), wheat germ agglutinin (WGA), and Hoechst. N = 15–24 tissues analyzed mechanically per condition; 1 mg/mL collagen concentration utilized; M indicates million; *n* = 7–13 tissues analyzed for histology with multiple regions averaged per tissue for each condition; the individual points represent single meso-ECTs analyzed; ** *p* < 0.01; *** *p* < 0.001, **** *p* < 0.0001.

To understand the functional consequences of increasing hiPSC-CM density, we measured the contractility of meso-ECTs by uniaxial tensile testing. Lower cell density meso-ECTs exhibited increased active stress generation at all tested strains (0–30% strain; Figure 5A and Supplemental Figure S3B–G). At the highest strain of 30%, active stress generation was significantly higher in the 5 M/mL condition (2.32 ± 0.24 mN/mm^2^) compared to both 15 M/mL and 30 M/mL conditions (15 M/mL: 1.29 ± 0.12 mN/mm^2^ and 30 M/mL: 0.79 ± 0.16 mN/mm^2^), as well as the higher 50 M/mL density condition (50 M/mL: 0.38 ± 0.06 mN/mm^2^) (Figure 5B). At day 7, the cross-sectional area (CSA) of the meso-ECTs showed increasing CSA correlating with cell density (5 M/mL: 0.10 ± 0.01 mm^2^; 15 M/mL: 0.19 ± 0.01 mm^2^; 30 M/mL: 0.36 ± 0.02 mm^2^; 50 M/mL: 0.71 ± 0.05 mm^2^) (Supplemental Figure S3A). Due to significant differences in ECT compaction and thus CSA used to normalize force for stress analysis, we also assessed raw force and force normalized by the number of input hiPSC-CMs. Interestingly, raw force generation was not statistically different between conditions, despite the increased number of hiPSC-CMs within the high-density meso-ECTs (Figure 5C). Normalization of force by hiPSC-CM input further revealed a significant increase in force generation per hiPSC-CM in the low cell density (5 M/mL: 1.31 × 10^−6^ ± 0.156 × 10^−6^ mN/hiPSC-CMs) compared to the higher cell density conditions (15 M/mL: 0.43 × 10^−6^ ± 0.04 × 10^−6^ mN/hiPSC-CMs; 30 M/mL: 0.27 × 10^−6^ ± 0.05 × 10^−6^ mN/hiPSC-CMs; 50 M/mL: 0.14 × 10^−6^ ± 0.02 × 10^−6^ mN/hiPSC-CMs) (Figure 5D). In addition to contractile stress magnitude, contractile kinetics as measured by upstroke velocity (Vup) and time to 50% and 90% relaxation (T50 and T90, respectively) were also quantified (Supplemental Figure S4). Vup was unchanged between density conditions, while small yet significant differences were detected in the time to relaxation when comparing 5 M/mL and 50 M/mL meso-ECTs across 10–25% strain, as expected from the higher force per cell in 5 M/mL conditions (Supplemental Figure S4C–F).

Because tensile mechanical analysis could only be performed on intact meso-ECTs at day 7, a video-based analysis of contractility using the software MUSCLEMOTION^®^ was implemented [24] (Supplemental Figure S5). This method enabled serial measurements for quantification of contractility throughout the culture to determine if there was a survival bias within the higher density conditions. Interestingly, the contractile amplitude measured through MUSCLEMOTION^®^ analysis of the meso-ECTs on day 7 in culture and force generation at 0% stretch measured by uniaxial tensile testing did not correlate well (R^2^ = 0.001, Supplemental Figure S5A). Additionally, it can be observed that contraction amplitudes, as measured through MUSCLEMOTION^®^, have a positive correlation with increasing cell density. This is likely due to the different contraction types observed in the meso-ECT density conditions during culture (Supplemental Figure S6), with lower density conditions contracted isometrically, thus maximizing force generation (uniaxial tensile stress) and limiting displacement (contraction amplitude measured through MUSCLEMOTION^®^ 2D displacement), while higher density conditions contracted isotonically, maximizing displacement.

In addition to analyzing the impact of cell density on the contractile properties of the meso-ECTs, we assessed the pacing frequency dependence of contraction, reporting a negative relationship for all meso-ECT conditions (Supplemental Figure S7). Most tissues were able to be paced up to 4 Hz, although the average pacing of the low 5 M/mL cell density condition averaged 3.5 Hz. Additionally, meso-ECTs, irrespective of density condition, began experiencing contractile alternans (defined as a >10% difference in beat-to-beat contractile amplitude) at 2.5 Hz pacing, which increased in magnitude with pacing as previously observed [19]. 

Histological staining was performed next to assess myofilament development and alignment (Figure 5E, Supplemental Figure S8). These results illustrate that sarcomere length was not significantly different between conditions (5 M/mL: 1.29 ± 0.03 µm; 15 M/mL: 1.27 ± 0.03 µm; 30 M/mL: 1.16 ± 0.04 µm; 50 M/mL: 1.24 ± 0.05 µm) (Supplemental Figure S8E). However, lower-density meso-ECTs (5 M/mL and 15 M/mL) displayed aligned sarcomeres throughout the tissue interior, while high-density meso-ECTs (30 M/mL and 50 M/mL) had limited sarcomere alignment only at the tissue edges. Further quantification of histological stains shows increased nuclear size of 5 M/mL meso-ECTs compared to 15 M/mL and 30 M/mL conditions (5 M/mL: 29.73 ± 1.29 µm^2^; 15 M/mL: 23.99 ± 0.67 µm^2^; 30 M/mL: 24.20 ± 0.78 µm^2^; 50 M/mL: 25.62 ± 1.57 µm^2^) (Supplemental Figure S8B). It is important to note that nuclear fragmentation, which is a hallmark of cells undergoing apoptosis and necrosis [48,49], was not found through the thickness of the meso-ECTs, suggesting that cellular viability was maintained. WGA and α-actinin content normalized by the number of nuclei was calculated, but no significant differences were found (Supplemental Figure S8C,D). 

Taken together, these results illustrate that increasing cell density within meso-ECTs compromises overall contractile stress generation, as measured through uniaxial tensile testing. Further, histological results corroborate that sarcomeres are underdeveloped with increasing hiPSC-CM density. In assessing meso-ECT function, decreasing cell density results in enhanced contractility both at the tissue and hiPSC-CM level with enhanced sarcomere organization. This is likely caused by the increased compaction and developed prestrain experienced by the hiPSC-CMs embedded within the low-density meso-ECTs. 

### 3.3. Increasing the Surface Area of ECTs Reveals Scale Impacts Formation and Cell Density Impacts Excitability without Inducing Arrhythmias 

The meso-ECTs utilized provided great insight into tissue formation and function at a small scale, which is useful for high-throughput in vitro assessment. However, in scaling up ECTs for preclinical and clinical applicability, the surface area is an important consideration to ensure coverage of the entire injured region of the myocardium. We thus scaled up our meso-ECTs (3 × 9 mm) into macro-ECTs (8 × 12 mm), which featured a larger surface area and additional PDMS posts to influence the final shape during compaction. For these experiments, we limited scope to a lower cell density condition of 15 M/mL, which was chosen as it has been a historical standard in our lab, as well as the high-density condition of 50 M/mL. Although the same collagen concentration of 1 mg/mL was initially used in macro-ECT fabrication, due to the high prevalence of macro-ECT breakage in both density conditions, the final collagen concentration was increased to 3.5 mg/mL for all experiments. This collagen concentration also balanced structural survival and elastic modulus with force generation when re-tested in the meso-ECTs (Supplemental Figure S9).

**Figure 6:**
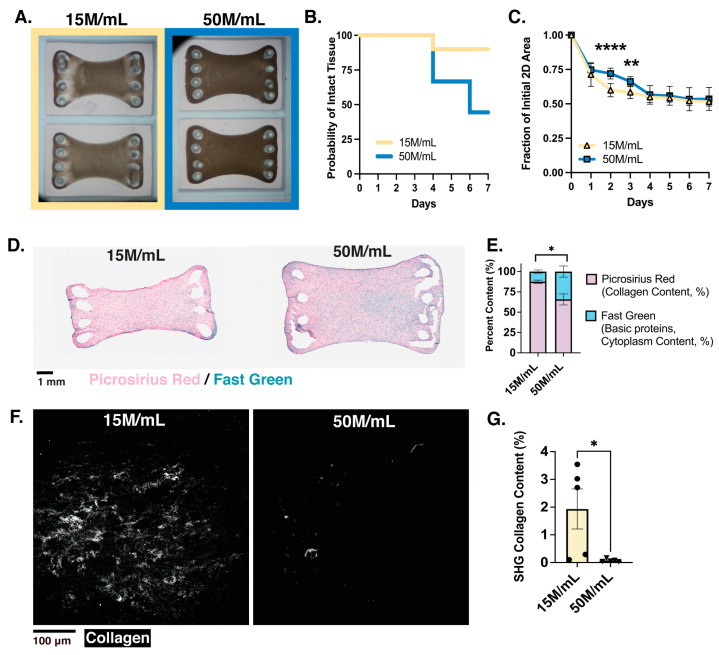
Macro-ECTs maintain dose dependence of tissue survival, collagen content, and organization but have more uniform compaction. (**A**) Brightfield images show macro-tissue compaction at day 7 (D7) of in vitro culture (mold size, 8 × 12 mm); (**B**) survival curve of percentage of intact tissues shows structural integrity begins to decline on day 4; (**C**) quantification of tissue compaction over 7-day culture; (**D**) representative histological staining of PRFG for each density condition; (**E**) quantification of the picrosirius red (collagen content) and fast green (basic proteins, cytoplasm content) per condition, measured by % of area analyzed; (**F**) representative images of SHG imaging showing organized collagen fibrils, measured by % area analyzed; (**G**) quantification of collagen content imaged through SHG for each condition. Collagen concentration of 3.5 mg/mL utilized; M indicates million; *n* = 9–10 tissues per condition analyzed for formation; *n* = 4–6 tissues analyzed histologically with multiple regions averaged per tissue for each condition; the individual points represent single macro-ECTs analyzed; * *p* < 0.5; ** *p* < 0.01; **** *p* < 0.0001.

Over 1 week of culture, macro-ECTs with densities of 15 M/mL and 50 M/mL formed and compacted (Figure 6A). Patterns of tissue structural survival in culture were comparable to meso-ECTs, with the 15 M/mL group exhibiting higher survival than the 50 M/mL group (Figure 6B). Macro-ECT structural survival was maintained in both groups until day 4 (15 M/mL: 90.00%; 50 M/mL: 66.67%), with continued fracture seen in the 50 M/mL group down to 44.44% at day 7. Unlike the meso-ECT system, high-density macro-ECT breakage occurred predominately at the posts, suggesting maintained structural internal integrity of the macro-ECT, which was not seen previously, likely due to the increased collagen hydrogel concentration utilized. Compaction of macro-ECTs stabilized by day 4 (15 M/mL: 0.55 ± 0.01; 50 M/mL: 0.57 ± 0.03, fraction of initial area), despite greater compaction at days 2–3 in the 15 M/mL group (Figure 6C).

ECM deposition and remodeling were evaluated using PRFG (Figure 6D,E) and SHG (Figure 6F,G). Similar to the meso-ECTs, increased cell density in the macro-ECTs correlated with decreased picrosirius red (collagen content) as measured by PRFG (15 M/mL: 77.14 ± 0.99%; 50 M/mL: 58.43 ± 6.57%, % area analyzed) as well as organized collagen measured by SHG imaging (15 M/mL: 1.93 + 0.72%; 50 M/mL: 0.072 + 0.02%, % area analyzed). Additionally, the 15 M/mL group exhibited a higher ratio of organized to unorganized collagen content (15 M/mL: 2.21; 50 M/mL: 0.11, measured as SHG signal normalized to PRFG collagen content). Normalization of collagen content to cardiac differentiation batch purity was performed by multiplying the differentiation purity (cTnT+ as assessed by flow cytometry), and quantification was performed to confirm its minimal effect (Supplemental Figure S10). Compared to the meso-ECT system, collagen content in the macro-ECTs of 15 M/mL and 50 M/mL densities assessed by PRFG increased by 52.0% and 45.2%, respectively, likely due to the 3.5-fold increased collagen hydrogel concentration used in tissue formation. However, organized collagen quantified through SHG imaging decreased in both conditions by 52.58% and 87.88% for the 15 M/mL and 50 M/mL conditions, respectively. The lack of structured collagen within the macro-ECTs may be due to the impact of the altered geometry on the internal tension generated within the tissue. These results suggest increased collagen concentration aids in stabilizing the structural integrity of macro-ECTs during the initial days of tissue formation, but not in prolonged culture. Furthermore, across the meso- and macro-ECT scales, differences in collagen remodeling are observed, likely due to geometric influences.

**Figure 7:**
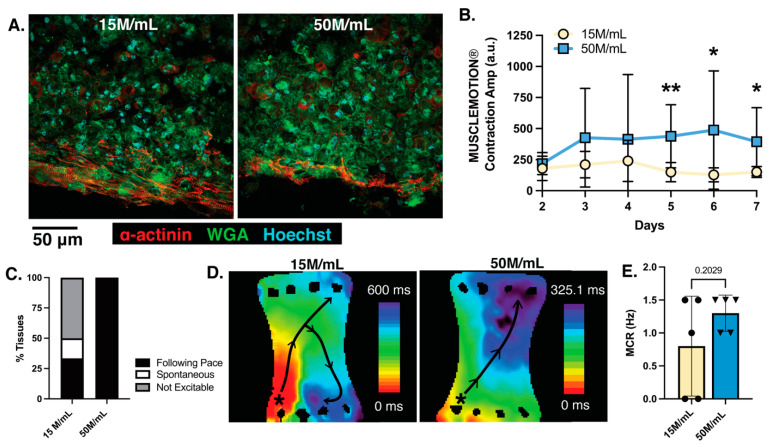
Increased cell density within macro-tissue format shows limited sarcomere organization but increased contractile amplitude and ability for electrical pacing with no arrhythmia generation. (**A**) Histological staining of α-sarcomeric actinin (α-actinin), wheat germ agglutin (WGA), and Hoechst; (**B**) video-based analysis of ECT contractility using the software MUSCLEMOTION^®^ to quantify contraction amplitude; (**C**) percent of tissues following 0.5 Hz point stimulation pacing during optical mapping; (**D**) heatmap of activation sequences generated from GCaMP calcium transient recordings for 15 M/mL macro-ECT (left) and 50 M/mL macro-ECT (right); (**E**) maximum capture rate (MCR) of macro-ECTs under field stimulation. Collagen concentration of 3.5 mg/mL utilized; M indicates million; *n* = 4–6 tissues analyzed with multiple regions averaged per tissue for each condition for histological analysis; *n* = 9–10 tissues per condition analyzed for contractility; *n* = 5–6 tissues per condition attempted for optical mapping, with pacing achieved in *n* = 2–5; the individual points represent single macro-ECTs analyzed; * *p* < 0.5; ** *p* < 0.01.

Macro-ECTs were next analyzed to determine the extent of structural development and function. Histological staining illustrated reduced sarcomere organization in the 50 M/mL condition as compared to the 15 M/mL condition (Figure 7A). However, in both conditions, aligned sarcomeres were limited to the tissue edge, with no significant differences found in sarcomere length (15 M/mL: 1.44 ± 0.04 µm; 50 M/mL: 1.33 ± 0.10 µm) (Supplemental Figure S11). Nuclei size as well as WGA and α-actinin content normalized by the number of nuclei was also calculated, but no significant differences were found between conditions (Supplemental Figure S11). Similar to the meso-ECTs, nuclear fragmentation, which is a hallmark of cells undergoing apoptosis and necrosis [48,49], was not found through the thickness of the macro-ECT, suggesting that cellular viability was maintained. Contractility as assessed by MUSCLEMOTION^®^ showed that the 50 M/mL macro-ECTs had significantly increased force generation compared to the 15 M/mL tissues at day 7, as measured by contraction amplitude (15 M/mL: 150.5 + 14.60 a.u.; 50 M/mL: 394.5 + 137.1 a.u., *p* = 0.019) (Figure 7B). 

A critically important aspect of ECT function is its electrical coupling to the host myocardium, which allows uniform propagation of action potentials to assist mechanical function by triggering synchronous contraction across the entire ECT in sync with the host. Arrhythmia generation from implanted hiPSC-CMs is a primary concern in recent literature, particularly with islands of injected (uncoupled) CMs [46,50,51], motivating our assessment of the electrophysiology in large surface area ECTs where reentrant arrhythmias may develop spatially. Macro-ECTs of 15 M/mL and 50 M/mL densities were optically mapped to assess calcium and voltage propagation, during which a stimulation electrode was placed on the corner of the ECTs to trigger a propagating excitation wave (point stimulation). One major difference between the density conditions was the ability of the macro-ECT to follow pace. All tested 50 M/mL macro-ECTs followed the 0.5 Hz point stimulation given. On the other hand, several 15 M/mL macro-ECTs were not excitable by pacing (0.3 mA, 2 ms duration) (50%) or exhibited spontaneous beating (16.66%) that was faster than the set pacing cycle length (Figure 7C). The activation maps of GCaMP calcium transients in the 15 M/mL condition displayed non-uniform propagation with conduction block and rotation, forming reentry. In the high-density 50 M/mL macro-ECTs, however, a smooth wavefront without rotation was present (Figure 7D). Voltage mapping was also performed (Supplemental Figure S12C), with local heterogeneities in the voltage mapping appearing smoothed in the calcium mapping, as expected from EC coupling kinetics. Conduction velocities in the area without conduction block were not significantly different in both GCaMP calcium transients (15 M/mL: 16.70 ± 13.74 x-direction, 24.42 ± 0.003 y-direction, 21.01 ± 4.75 xy-direction; 50 M/mL: 25.55 ± 9.55 x-direction, 26.73 ± 7.11 y-direction, 17.52 ± 3.72 xy-direction) and action potentials (15 M/mL: 8.39 ± 4.64 x-direction, 15.62 ± 2.1 y-direction, 11.74 ± 1.56 xy-direction; 50 M/mL: 5.83 ± 1.68 x-direction, 18.86 ± 4.17 y-direction, 9.892 + 1.30 xy-direction) (Supplemental Figure S12). In measuring propagation velocity, point stimulation was used to mimic the excitatory signal that the macro-ECT would likely receive from neighboring host cells if it was engrafted and coupled into a host heart. However, field stimulation was also performed to determine the maximum capture rate that could be achieved. In this case, the high-density 50 M/mL macro-ECTs trended toward higher stimulation rates as compared to the 15 M/mL macro-ECTs (15 M/mL: 0.80 ± 0.34 Hz; 50 M/mL: 1.30 ± 0.12 Hz, *p* = 0.209). 

Together, our results show that cell-dense macro-ECTs have a greater ability to follow electrical pacing with little to no concern for arrhythmia generation in vitro. In scaling up the surface area of ECTs, spatial consideration of both contractility and electrical propagation showed encouraging functional results in the highest 50 M/mL macro-ECTs.

### 3.4. Clinical Scale Mega-ECT Enables Delivery of 1 Billion hiPSC-CMs to the Chronically Ischemic Swine Heart

The primary goal of this study was to assess ECTs through the lens of clinical translation. Therefore, we further scaled up our fabrication and workflow to accommodate an ECT of clinically relevant cell dose and size. Up to this point, we had utilized smaller-scale ECTs to elucidate how cell density impacts tissue formation and function, reporting some compromises (i.e., decreased compaction and sarcomere organization) and benefits (i.e., reduced arrhythmia) that exist when fabricating cell-dense planar ECTs. Because of this delicate balance between tissue function and density, it becomes necessary to prioritize which properties are essential for therapeutic application, which is why we chose to prioritize cell dose. In considering ECTs for regenerative applications, we hypothesize that compromises in tissue function in vitro immediately after formation can be overcome through in vivo maturation of the ECT after implantation, which we [52] and others [53,54,55] have reported. Thus, our goal was to embed 1 billion hiPSC-CMs within our mega-ECT using a density of 50 M/mL. 

**Figure 8:**
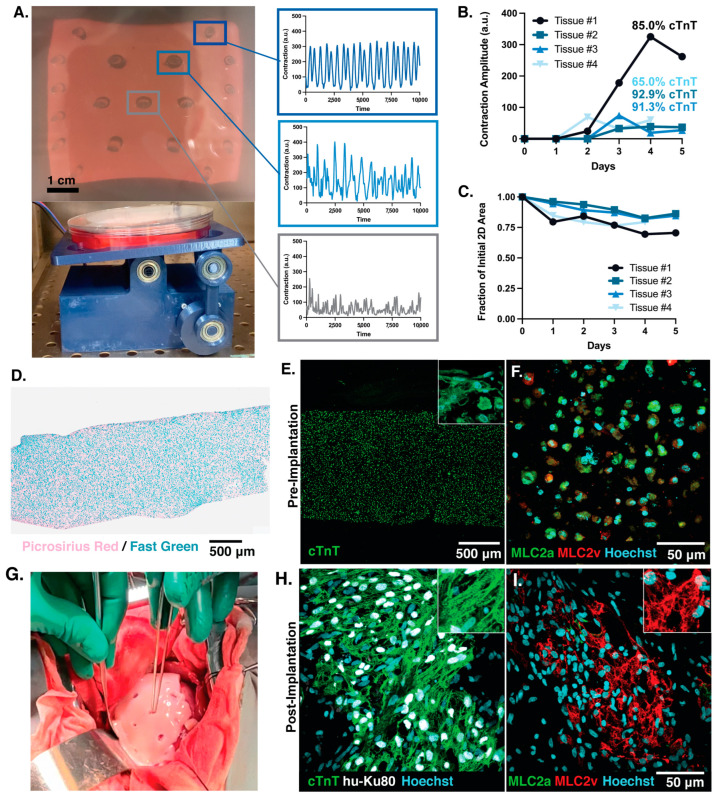
Scale-up to clinical-sized engineered tissue allows for the delivery of 1 billion hiPSC-CMs within a single tissue. (**A**) Brightfield images illustrating mega-tissue compaction during in vitro culture (mold size, 65 × 75 mm) and tissue under dynamic culture on a rocker; (**B**) video-based analysis of ECT contractility using the software MUSCLEMOTION^®^ to quantify contraction amplitude at the external post of the mega-ECT; (**C**) quantification of tissue compaction over in vitro culture; (**D**) representative cross-sectional PRFG stain from the middle of the mega-ECT pre-implantation; (**E**) cross-section histological staining of the full transverse of the middle portion of the mega-ECT with hiPSC-CM marker, cTnT pre-implantation; (**F**) histological staining of myosin light chain 2, specifically MLC2a which represents the immature isoform and MLC2v which represents the more mature isoform pre-implantation. (**G**) Implantation of mega-ECTs on epicardial surface of swine model of chronic ischemia model; (**H**) histological staining of mega-ECT post-implantation with cTnT, human-specific nuclear marker (hu-Ku80), and Hoechst with section taken from the anterior-lateral basal region of the heart; (**I**) histological staining of mega-ECT post-implantation with MLC2a, MLC2v, and Hoechst. *n* = 4 mega-ECTs fabricated and assessed for structural survival, compaction, and contractility with section taken from the anterior-lateral basal region of the heart. 50 M/mL hiPSC-CM density and 3.5 mg/mL collagen concentration utilized; M indicates million; *n* = 4 mega-ECTs fabricated; *n* = 2 implanted mega-ECTs in swine model; *n* = 1 analyzed for histology with multiple regions averaged for all quantification.

Because of this massive cell quantity, an important aspect to consider during the manufacturing of the mega-ECT was the clinical environment in which this therapy would be employed. The left ventricle (LV) lumen of the human heart at its midpoint is roughly 40 mm in diameter and 90 mm long during diastole, as measured by contrast-enhanced computed tomography [56]. Estimating the LV as a semi-ellipsoid, the maximum surface area of the LV can be calculated to be ~10,287 mm^2^. Assuming the surgical field of view cuts the LV in half, there is a viable surface area for implantation of less than ~5144 mm^2^. We thus sought to engineer our mega-ECT to maximize this implantable surface area, as it allowed us to: (1) cover the infarct as well as part of the healthy myocardium; (2) minimize the necessary cell density; and (3) minimize tissue thickness (measuring ~1–1.5 mm after compaction). To achieve this, a PDMS mold of dimensions 75 × 65 mm, and therefore a working surface area of 4875 mm^2^, was generated and used for casting the ECT. Utilizing a cell density of 50 M/mL in 20 mL with 3.5 mg/mL collagen hydrogel, we fabricated mega-ECTs containing 1 billion hiPSC-CMs (Figure 8A). 

To promote nutrient diffusion within the tissue, we employed dynamic culture using a custom rocking plate adapted from NIH 3D [57], which has been reported in the literature to support the culture of thick (~1.25 mm) ECTs [58] (Figure 8A). After 2–3 days of dynamic culture, we observed beating throughout the tissue as analyzed by MUSCLEMOTION^®^ (Figure 8A,B). Tissue compaction at day 4 was calculated to be 0.78 ± 0.03 (fraction of initial area), which was decreased compared to the 50 M/mL group of the macro-ECTs (0.57 ± 0.03) and meso-ECTs (0.40 ± 0.02) (Figure 8C). In scaling up, the location of PDMS posts was updated to include internal posts, which stabilized the tissue during culture and further promoted nutrient diffusion. Post spacing was informed by our prior work [20] in order to minimally disrupt electrical propagation. For the evaluation of our mega-ECTs during culture, we found these internal posts essential, as tissue compaction away from the posts allowed us to visualize contractility throughout the tissue. Immunohistochemical analysis of the mega-ECT at day 5 revealed cells throughout the tissue thickness with no evidence of necrotic patches (Figure 8D,E, Supplemental Figure S13B). We hypothesize the decreased cell density of the mega-ECT as compared to native myocardium (estimated as ~10^8^–10^9^ cell/cm^2^, [59]) as well as the dynamic culture conditions implemented that allowed for oxygen and nutrient diffusion throughout the thickness of the tissue. PRFG staining was performed with an average collagen content measured to be 84.67 ± 0.69% (Supplemental Figure S13C,D) as well as further quantification from histological staining (Supplemental Figure S13E,H). Unlike the meso- and macro-ECTs, we did not observe sarcomere elongation and organization. We hypothesize that this lack of organization, as well as the different contractility amplitudes observed throughout the tissue, may be a consequence of the decreased compaction and strain felt by the embedded hiPSC-CMs in this larger format. 

As a next step in translation, the mega-ECTs were assessed in a swine model of chronic myocardial ischemia. In this surgical model, chronic ischemia was induced by placing an ameroid constrictor around the left circumflex artery (LCX, Figure 2B). The slow swelling of the constrictor reduced blood flow and gradually induced ischemia, as opposed to rapid occlusion (often used experimentally), to reflect clinically relevant cases in patients with coronary artery disease. After 4 weeks of ischemia, the mega-ECTs were implanted on the epicardial surface of the swine heart (*n* = 2) to assess technical feasibility. An aggressive immunosuppression regimen [45,46] was administered daily starting one week prior to ECT implantation to minimize xenograft rejection. The mega-ECTs were structurally robust to allow for successful transportation, surgical handling, and implantation onto the epicardial surface (Figure 8G) via sutures. We noted, however, that the mega-ECTs would benefit from a further increase in mechanical integrity to withstand the strong contractile forces of the beating swine heart. Unfortunately, one animal experienced surgical complications (presumed blood clot), resulting in premature death.

Histological analysis to determine hiPSC-CM engraftment was performed 4 weeks after implantation in the surviving swine, revealing persistent human grafts, assessed with the human nuclear marker Ku80 (Figure 8H, located in the anterior-lateral basal region of the heart). Surviving human grafts featured cardiomyocytes with highly organized sarcomere structures as well as high levels of the mature myosin light chain, MLC2v, compared to the more immature isoform, MLC2a. Comparing this endpoint histology (Figure 8I, located in the anterior-lateral basal region of the heart) to that of the pre-implant control (Figure 8F) suggests that significant in vivo maturation of the hiPSC-CMs occurred. In this chronic ischemia model, the scar morphology of the swine successfully implanted with the mega-ECT showed interstitial, diffusive fibrosis through immunohistochemical staining of the LV (Supplemental Figures S14–S17). Heart rate was monitored daily throughout the study, confirming no occurrence of tachyarrythmias (Supplemental Figure S18). Together, our results show successful delivery and engraftment of hiPSC-CM patches in a swine model of chronic myocardial ischemia with no arrhythmia generation. To our knowledge, this is the first implantation of 1 billion hiPSC-CMs within a single ECT in a swine model of MI injury and the first to utilize the chronic ischemia model with PSC-CM delivery. 

## 4. Discussion

The goal of cell-based cardiac regeneration therapies is to remuscularize the heart and subsequently improve cardiac function after injury. Preclinical models have repeatedly shown engraftment of PSC-CMs in diseased hearts [46,52,60,61,62], with recent work confirming improved contractile function with engraftment [12,50,58,63,64,65]. Like any treatment, however, dose is a key factor impacting therapeutic efficacy. Work by Querdel et al. reports a dependence of remuscularization on the dose of hiPSC-CMs implanted in vivo [12]; yet, the appropriate cell dose required for maximal functional benefit after cardiac injury remains unknown. In the case of MI, it is likely this therapeutic dose is on a spectrum, dependent on factors such as disease severity (infarct size, location, etc.), progression of healing (remodeling, dilation, time between injury and intervention, etc.), as well as patient demographics (age, anatomy, response to treatment, comorbidities, co-therapies, etc.). In this way, there is great value in being able to tune cell dose within ECTs to accommodate patient variability and disease state. 

Despite the importance of scaling up ECTs in terms of cell dose to maximize therapeutic impact as well as size for clinical relevance, the design parameters have not been adequately defined for manufacturing ECTs that can accommodate up to 1 billion CMs for preclinical testing (Table 1) or clinical trials (Table 2). In this study, we sought to enhance the field’s fundamental understanding of manufacturing dose within ECT to define the design space for engineering implantable ECTs with varying hiPSC-CM density by identifying the criteria, constraints, and challenges of this system. By fabricating ECTs across three length scales, we were able to study different key aspects of ECT formation and function, demonstrating that scaling up ECTs is a nontrivial and nonlinear challenge. Beginning with meso-ECTs, we investigated aspects of formation and function in a higher throughput manner. The wide range of cell densities (5 M/mL, 15 M/mL, 30 M/mL, 50 M/mL, and 75 M/mL) chosen allowed us to compare our results to current literature (~5–15 M/mL; Table 1), while also defining the design limits of the system. High densities were essential to test as they allowed the scale-up process to reach 1 billion hiPSC-CMs within a single ECT of reasonable size for implantation on the adult heart. For example, the lowest density condition of 5 M/mL would require a volume of 200 mL to contain 1 billion hiPSC-CMs, an order of magnitude higher than the volume used in our mega-ECT, which is unrealistic to maintain an appropriate tissue surface area and thickness. Therefore, we evaluated hiPSC-CM densities up to 75 M/mL and determined that 50 M/mL is a workable solution for creating human-sized ECTs. Through this upscaling effort, we successfully embedded 1 billion hiPSC-CMs within a clinically sized ECT (6.5 × 7.5 cm). There are few studies investigating scaling ECT to clinically relevant size (>3 × 3 cm), and these are often limited to low PSC-CM doses (4 M [58] and 10 M [61]). In the latter case, for example, ECTs were upscaled from a surface area of 7 × 7 mm/0.5 × 10^6^ hiPSC-CMs to 15 × 15 mm/2M hiPSC-CMs and further to 3.6 × 3.6 cm/10 M with an estimated density of ~0.2–0.3 M hiPSC-CMs/mL, which is more than two orders of magnitude lower than the 50 M hiPSC-CMs/mL utilized in our current study. Fabrication of robust, cell-dense ECTs is essential for encapsulation and delivery of therapeutically impactful cell numbers, as well as robust surgical handling and suturing of the tissue during implantation.

Through a holistic examination of our results, we identify both hiPSC-CM density (Supplemental Figure S19A) and scale (Supplemental Figure S19B) as important factors that influence ECT formation and function in vitro. Within our small-scale meso-ECTs, density impacted the environment within the tissues, leading to changes in the structural and mechanical development of the tissue, aligning with other reports in the literature. For example, Shadrin et al. varied the hiPSC-CM dose from just 0.5 M to 1 M within ECTs, finding decreased stress generation (21.0%) as well as force per CM (43.7%) and decreased conduction velocity (212.6%) [61]. Biomechanical cues such as passive stretching and loading have also been shown to improve ECT structural organization, contractility, and matrix deposition [68,69,70]. In upscaling to the planar macro-ECT, mechanical cues shifted compaction dynamics as maintaining a maximal surface area of the tissue became more important. Unlike the meso-ECTs, both compaction and mechanical function of the macro-ECTs in vitro converged regardless of density, suggesting the significant influence of tissue scale on tissue formation and function. Further functional benefits were also noted in the cell-dense macro-ECT condition. For example, of significant clinical importance and translation is the ability to study how the action potentials and calcium transient signals propagate throughout the larger surface area of upscaled ECTs. Arrhythmia generation from PSC-CMs is of great clinical relevance, as recent studies have shown that injecting PSC-CMs has resulted in life-threatening arrhythmia generation in preclinical models, hypothesized to be due to the spontaneous excitation of the implanted CMs [46,50,51]. At the macro-ECT level, however, increased hiPSC-CM density enabled better pacing control as well as uniform wavefront propagation, suggesting a reduced risk of arrhythmia. These findings encouraged us to fabricate an even larger mega-ECT composed of 1 billion hiPSC-CMs for implantation in a large animal model of chronic myocardial ischemia. Upon implantation of our mega-ECT in vivo on the epicardial surface of the swine heart, significant maturation of hiPSC-CMs occurred over the next 4 weeks. This data supports the notion that ECTs for applications as regenerative therapies may need to prioritize cell quantity and structural integrity of the tissue while allowing functional maturation to occur after implantation. This study has some limitations, such as the few mega-ECTs that could be fabricated due to the large quantity of hiPSC-CMs needed for each; the high cost associated with differentiation of hiPSC-CMs and their labor-intensive maintenance in culture; the lack of culture under clinical-grade GMP conditions; as well as the preclinical experiments utilized as feasibility of engraftment, rather than powered for functional outcome.

In considering in vitro fabrication and metrics of ECT function for in vivo implementation, we revealed fundamental insights that inspire further innovation. Through scaling up, we identified several challenges throughout the ECT manufacturing process that must be overcome with creative engineering to fabricate cell-dense ECTs with high clinical therapeutic potential.

### 4.1. Challenge #1: Structural Integrity for Surgical Handling of Cell-Dense ECTs

One of the greatest challenges in engineering large, cell-dense cardiac tissues using hydrogels to establish the physiological ECM microenvironment is their low structural integrity. Above all other properties, structural integrity is fundamental for fabricating ECTs for regenerative therapies, as it ensures the tissue will remain intact throughout culture, transportation, and surgical handling. Hydrogels are used extensively in engineering cardiac tissue, as they provide necessary components of the native tissue environment and enough structural and mechanical support to maintain embedded cells while they remodel and deposit their own ECM. However, hydrogels themselves are mechanically weak, with elastic moduli for collagen (0.5–2 mg/mL) and fibrin (10–20 mg/mL) hydrogels of relevant concentration for tissue engineering reported to range from 0.5–3.73 kPa [23]. In cell-dense ECTs, where remodeling of the environment is limited, engineering-enhanced tissue structural integrity will likely be required.

In this work, we made the design decision to upscale our ECTs by utilizing collagen hydrogels because of their ability to support hiPSC-CMs, which are very challenging cells to grow and maintain. Reflecting on our results, we hypothesize that there exist promising avenues that can improve the structural integrity and handle-ability of cell-dense ECTs. First, ECT structural integrity could be increased by promoting intrinsic ECM deposition of the embedded cells, such as through stretch [68,69] or increased fibroblast content [19]. A second approach involves extrinsically stabilizing the tissue using robust scaffolding. Cardiovascular scaffolds for engineered tissues are in no short supply [71]. Passive scaffolds on the heart after injury alone have even been shown to reduce ventricular diameter and improve cardiac ejection fraction [72,73,74,75]. Interestingly, such reinforcement has also been shown to decrease hypertrophy and improve contractility of resident CMs through mechanical unloading [76]. Incredible advancements in these technologies have allowed scaffolds to be fabricated with precisely tuned mechanics [77,78,79,80,81,82], anisotropy [21,83,84], biomimetic properties [21,77,85,86,87,88] and biodegradability [89,90,91,92,93]. Current work in the field exploring beneficial properties for tissue formation and function can be applied to increase ECT structural stability while also promoting engraftment of hiPSC-CMs in vivo.

### 4.2. Challenge 2: Maturation State of PSC-CMs

Traditionally, a primary goal in tissue engineering has been to generate a biomimetic tissue in vitro with similar compositional, structural, and functional characteristics to that found in the native body. In the case of the heart, decades of research have been aimed at enhancing the structural sarcomere organization, contractile stress generation, electrical conduction velocity, and metabolism of PSC-CMs to more closely match those of native adult myocardium [94,95,96]. Such efforts have accelerated the use of PSC-CMs and miniaturized engineered cardiac tissue platforms for a variety of applications, such as drug testing and disease modeling, where targeted PSC-CM maturation is required to mimic adult myocardium and/or observe pathological phenotypes. Yet, functional outputs of even the “most mature” engineered cardiac tissue are still lower than what is reported for the native human myocardium (elastic modulus: ~20 kPa; twitch stress: ~17 kPa; conduction velocity: ~80 cm/s) [97]. In regenerative engineering applications, we must reconsider this widely adopted “requirement” for mature PSC-CMs as our understanding of in vivo regeneration grows. In this work, we suggest that prioritizing cell density for transplant requires a reassessment of in vitro benchmarks in order to reach longer-term in vivo goals. This is supported by other studies in the field that suggest maturation of implanted PSC-CMs or engineered tissue prior to implantation may not be an appropriate measure of a quality tissue product. Recent work in the field points to the immaturity of PSC-CMs as providing protection from ischemia upon implant to promote engraftment [63,98]. In vitro, Peters et al. report that metabolically matured hiPSC-CMs demonstrated decreased mitochondrial respiration and increased cell death after hypoxia exposure [98]. In vivo, Weinberger and Eschenhagen directly compared immature and matured hiPSC-CM within an ECT using a cryo-injury guinea pig model of MI, finding that the immature ECTs had higher hiPSC-CM engraftment as well as increased improvement in left ventricular function after 4 weeks [63].

Thus, an emerging question we are reconsidering is: what metrics are “desirable” for ECTs that are designed specifically for regenerative applications? Learning from our work as well as that of others, the standards to which we define success for the application of ECTs as a regenerative therapy are likely not entirely going to be based on how well these systems mimic the native adult myocardium. When considering the harsh, inflamed, and scar-filled environment of the post-ischemic ventricle, it makes sense that the plasticity of immature hiPSC-CMs may actually be beneficial to promote engraftment and remuscularization, which is the ultimate goal of these regenerative therapies. However, specific aspects of hiPSC-CM maturation are desirable for regenerative applications. For example, there is valid concern about patient safety associated with arrhythmia initiation from engrafted PSC-CMs, and if the immature electrophysiology of PSC-CMs is contributing to life-threatening arrhythmia generation in preclinical models [46,50,51] then an appropriately “mature” or quiescent electrophysiological profile is warranted prior to PSC-CM implant. Work by Dhahri et al. reports that when hiPSC-CMs are structurally but not metabolically matured in vitro on a soft PDMS substrate, the cells have enhanced electromechanical integration, less proarrhythmic behavior, and a greater therapeutic effect on contractility in a guinea pig model of MI [99]. This work highlights the vital importance of continuing to understand the developmental biology behind PSC-CM maturation in order to better target specific pathways associated with different measures of maturation and predict their impact on PSC-CMs as regenerative therapies. Tuning such maturation pathways could improve engraftment, thus decreasing the necessary cell dose to achieve therapeutic benefit, and simultaneously reducing arrhythmia risk and other side effects.

### 4.3. Challenge 3: Delivery of PSC-CMs

The majority of current clinical trials inject PSC-CMs directly into the ventricular wall. This approach has clinical advantages, as injection into the LV wall is minimally invasive compared to ECT implantation on the epicardial surface of the heart. Additionally, dose can be easily modulated in this delivery system by increasing the number of injections and/or volume of PSC-CMs injected. However, this therapy suffers from reduced cell retention [100,101,102]. PSC-CMs injected into the myocardium can be lost due to leakage from the injection site or flushed away from the target site by local bleeding or contracting myocardium. Indeed, Terrovitis et al. report that when cells are injected into the contracting and perfused myocardium, less than 20% are retained 1 hr post-injection, as assessed by in vivo PET in a rat permanent ligation MI model [103]. In contrast, when cells were injected after cardiac arrest, in which there was no contraction or coronary flow, retention was increased to more than 70%. For diseases such as MI, the vascularization and contractility in the ischemic region of the LV are compromised, which may increase cell retention. However, more work is necessary to evaluate retention more accurately in different clinical conditions of MI and to estimate the proper CM doses necessary to reach therapeutic efficacy.

To overcome cell retention problems, many groups, including ours, utilize ECTs, which are also currently in clinical trials. Compared to injections, ECTs provide greater control over PSC-CM delivery to the infarcted area via suturing onto the epicardium. ECTs provide increased mechanical reinforcement to the LV to help prevent dilation and increased design control over the microenvironment to support PSC-CMs prior to implantation, such as pre-vascularization [52] and/or mechanical reinforcement [21,82]. For single-entity tissues, PSC-CMs are cultured to promote syncytium formation with electro-mechanical cohesion, which may help to overcome arrhythmogenesis associated with islands of PSC-CMs reported with injection [46,50,51]. Yet, further innovation is necessary to directly electrically couple ECTs with the host heart to synchronize contraction and maximize recovery of cardiac function. With upscaled cell-dense ECTs, electromechanical coordination throughout the tissue remains an active research pursuit. We believe spatial heterogeneity in prestrain development through the tissue causes non-uniform excitation and contraction, and more evaluation and iteration are needed in the field using ECTs with a large surface area to determine the extent and impact of functional heterogeneity. Although we have developed a single-entity ECT to deliver up to 1 billion PSC-CMs, modular approaches using multiple stacked ECTs are being used to deliver higher doses of PSC-CMs [104]. Although this approach does not support full tissue syncytium formation and requires more technical manipulation of ECTs in vitro and/or during implantation in vivo, it comes with some advantages. First, risk is reduced in the fabrication and culture of each tissue. Second, it allows for greater design control within the thickness of the tissue to incorporate therapeutics that would benefit from being uncoupled from ECT culture, such as biomaterials containing growth factors that would otherwise release during in vitro culture [60]. Third, according to our data, decreasing cell densities may improve hiPSC-CM function prior to implant if this proves to be functionally beneficial for regeneration.

### 4.4. Challenge 4: Treatment of Comorbidities

Ischemic injuries to the heart such as MI often occur in a complex environment. Many pathophysiological components of MI, such as inflammation, microvascular dysregulation, scarring, wall thinning, ventricular remodeling, mitral regurgitation, etc., likely contribute to the extent and type of LV remodeling as well as HF development. Although the most fundamental of these components is the loss of functional cardiomyocytes, which cell-based therapies aim to address, several other components likely impact the efficacy of implanted cells to restore contractility. For example, recent work from our group illustrates that in vitro perfusion of ECTs containing patterned vasculature improved in vivo engraftment as well as intra-implant vascular organization [52]. Another concern with the implantation of allogeneic cells is the harsh immune suppression required to support cell engraftment without rejection. Recent work by Deuse and Hu et al. details the engineering of hypoimmune induced pluripotent stem cells (iPSC) through the inactivation of major histocompatibility complex class I and II and over-expression of CD47 [105]. In this way, hypoimmune iPSCs can be differentiated and evade immune rejection upon implantation in fully immunocompetent recipients [106]. Such multi-faceted approaches to treating the diseased heart may help to improve the engraftment of PSC-CMs as well as their function within the diseased myocardium and similarly reduce the dose of PSC-CMs necessary to be therapeutically impactful.

## 5. Conclusions

As clinical trials for PSC-CM regenerative cardiac therapies shift from feasibility and safety to pivotal studies of efficacy, cell dose is a key factor impacting both the efficacy and safety of these therapies. In this work, we leveraged scale-specific aspects of ECT formation and function to define biomanufacturing parameters for creating high-density ECTs. With high-throughput small-scale ECTs (meso-ECTs, 3 × 9 mm), we report the significant impact of hiPSC-CM density on tissue formation and function over 7 days of culture in vitro, allowing us to narrow our range of tested cell densities and hydrogel composition in order to support ECT formation. Scaling up to a larger, planar surface area (macro-ECTs, 8 × 12 mm), we additionally evaluated the impact of hiPSC-CM density on electrical signal propagation and tissue arrhythmogenesis after 7 days of in vitro culture, finding cell-dense macro-ECTs were able to follow point stimulation pacing without arrhythmogenesis better than the low-density condition. Finally, we fabricated and implanted a single mega-ECT (65 × 75 mm) composed of 1 billion hiPSC-CMs into a swine model of chronic myocardial ischemia. We successfully demonstrated the technical surgical feasibility of this therapeutic approach, as well as reported engraftment and maturation of iPSC-CMs without incidence of arrhythmia over 4 weeks in vivo. Through this work, we have identified hiPSC-CM density as well as ECT size and shape as vital considerations in scaling up to clinically relevant cell doses. Taken together, this study refines the many variables involved in ECT biomanufacturing at a clinically relevant scale that influence ECT formation and function, considers what may be important for in vivo vs. in vitro endpoints, and identifies the ways to leverage these variables to create ECT therapies with high translation potential for heart regeneration.

## Figures and Tables

**Table 1:** Doses of hiPSC-CMs within ECTs reported in current preclinical research for cardiac regenerative therapies.

Research Group	Dose(s)/ECT	Dimensions	Experiments
Eschenhagen and Weinberger [12,63]	450 M (hiPSC-CMs)	50 × 70 mm	In vivo: healthy swine heart Successful engraftment of hiPSC-CMs
	4.5 M, 8.5 M, 12 M, 15 M (hiPSC-CMs)	15 × 25 mm	In vivo: guinea pig cryoinjury model Cell dose dependence on remuscularization of scar Improvement in LV FAS at high dose (12 M) Importance of hiPSC-CM maturity for engraftment
Zhang [58]	4 M/ECT 8 M/2-ECTs (hiPSC-CMs)	40 × 20 mm	In vivo: swine I/R model of MI 2 ECTs implanted over infarct Improved EF compared to sham and non-cellular implants Decreased LVEDV and infarct size compared to sham and non-cellular implants
Bursac [61]	0.5 M/1 M (hiPSC-CMs)	7 × 7 mm	In vitro/in vivo: Increased stress generation, force per CM, velocity of action potential at lower cell density No adverse effect on host electrical function
	2 M/10 M (hiPSC-CMs)	15 × 15 mm 36 × 36 mm	In vitro: Scale up for proof of concept
Coulombe [60]	7–10 M (hiPSC-CMs)	18 × 14 mm	In vivo: rat I/R model of MI Trend illustrating improved viable engrafted area of ECTs loaded with proangiogenic factors Correlated with improved vascularization of ECT as well as infarct area with proangiogenic loading of ECTs Improved FS of ECTs with angiogenic factor loading compared to sham
Keller [66]	2.64–5.28 M (hiPSC-CMs)	15 × 15 mm	In vivo: rat model of MI Improved EF, CO, and regional LV radial and longitudinal strain at 4 weeks
	10.56 M (hiPSC-CMs)	30 × 30 mm	In vitro: Scale up for proof of concept
Zimmerman [64]	2.5 M/ECT 12.5 M/5-ECT (Neonatal rat CMs)	~5 × 10 mm	In vivo: rat PL model of MI Generating multiloop ECTs by stacking 5 single loop ECTs Induced systolic wall thickening Improved FAS compared to controls

MI = myocardial infarction, LV = left ventricle, I/R = ischemia-reperfusion, PL = permanent ligation, FAS = fractional area shortening, FS = fractional shortening, LVEDV = left ventricle end diastolic volume, EF = ejection fraction, CO = cardiac output; M = million.

**Table 2:** Doses of hiPSC reported in current human clinical trials for cardiac regenerative therapies.

Trial Number	Dose	Disease	Description	Phase	Start/End
NCT03759405	Not specified	CHF	Autologous iPS-CMs via vein transplantation	2–3	December 2022/ December 2024
NCT03763136	200 M	CHF	Injection of allogenic hPSC-CM during coronary artery bypass surgery	1–2	October 2021/ July 2023
NCT04396899	Not specified	HFrEF (EF < 35%)	Engineered heart muscle	1–2	February 2020/ October 2024
NCT05068674	10 M/150 M/300 M	Chronic ischemic LV dysfunction	Dose tolerance study of hESC-CMs	1	March 2022/October 2025
NCT04982081	100 M/400 M	Congestive HF, CVD, dilated Cardiomyopathy	hiPSC-CMs catheter injection	1	September 2021/ July 2023
NCT04696328/ jRCT2053190081 [67]	(3) sheets of 33 M /sheet	Ischemic cardiomyopathy	Allogenic iPSC-CM within a cell sheet	1	December 2019/May 2023

CHF = chronic heart failure, CVD = cardiovascular disease, HFrEF = heart failure with reduced ejection fraction, MI = myocardial infarction/ischemia, LV = left ventricle, EF = ejection fraction; M = million.

## Data Availability

Data will be made available upon request.

## Supplementary Materials

The following supporting information can be downloaded at: https://www.mdpi.com/article/10.3390/bioengineering10050587/s1. Supplemental Table S1: Antibodies used in immunohistochemical staining; Supplemental Table S2: Density of Meso-ECTs input versus compacted; Supplemental Figure S1: Representative histological staining of picrosirius red/fast green (PRFG) for acellular 1 mg/mL collagen constructs; Supplemental Figure S2: Meso-ECT collagen content quantified by picrosirius red/fast green (PRFG) and second harmonic generation (SHG) imaging normalized by cardiac differentiation batch purity; Supplemental Figure S3: In depth active mechanical analysis of contractile magnitude for meso-ECTs; Supplemental Figure S4: In depth active mechanical analysis of contractile kinetics for meso-ECTs; Supplemental Figure S5: Video-based analysis of active mechanics of meso-ECTs using MUSCLEMOTION^®^ software; Supplemental Figure S6: MUSCLEMOTION^®^ analysis of meso-ECTs; Supplemental Figure S7: Stress-frequency response of contractile magnitude and kinetics for meso-ECTs; Supplemental Figure S8: Quantification of meso-ECTs histological staining using Hoechst (nuclear marker), WGA (cell surface) and α-actinin (sarcomere marker); Supplemental Figure S9: Increasing collagen content in meso-ECTs; Supplemental Figure S10. Macro-ECT collagen content quantified by picrosirius red/fast green (PRFG) and second harmonic imaging normalized by cardiac differentiation batch purity; Supplemental Figure S11. Quantification of macro-ECTs histological staining using Hoechst (nuclear marker), WGA (cell surface) and α-actinin (sarcomere marker); Supplemental Figure S12. Optical mapping of macro-ECTs; Supplemental Figure S13. Mega-ECT fabrication and structural analysis; Supplemental Figure S14. Hematoxylin and eosin (H&E) staining of explanted swine heart; Supplemental Figure S15. Picrosirius red/fast green staining of explanted swine heart; Supplemental Figure S16. Aniline blue collagen staining of explanted swine heart; Supplemental Figure S17. Electrophysiology analysis of swine model throughout study. Supplemental Figure S18. Electrophysiology analysis of swine model throughout study. Supplemental Figure S19. Direct comparison of quantified metrics for cell density and ECT size.
