# Supplementary material for: One Billion hiPSC-Cardiomyocytes: Upscaling Engineered Cardiac Tissues to Create High Cell Density Therapies for Clinical Translation in Heart Regeneration
Source: Bioengineering (Basel). 2023 May 13;10(5):587. doi: 10.3390/bioengineering10050587 (PMC10215511; doi:10.3390/bioengineering10050587)
Supplement: Supplementary file 1 [file bioengineering-10-00587-s001.zip › bioengineering-2350488-supplementary.pdf]

Supplemental Tables/Figures:

Supplemental Table S1: Antibodies used in immunohistochemical staining.

Primary/Secondary Antibody	Dilution	Catalog Number
Mouse monoclonal anti- α-sarcomeric actinin	1:500	MilliporeSigma; A7811-0.2ML
Mouse monoclonal anti-cTnT	1:100	Invitrogen; MA5-12960UL
Rabbit polyclonal anti-MLC2v	1:100	ProteinTech; 10906-1-AP
Mouse monoclonal anti-MLC2a	1:100	Synaptic Sytems; 311 011
Bisbenzimide H 33342 trihydrochloride (Hoechst)	1.5 ug/mL	MilliporeSigma; B2261-100MG
Goat anti-mouse/rabbit Alexa Fluor 488	1:250	Invitrogen; A1100/A11008
Goat anti-mouse/rabbit Alexa Fluor 594	1:250	Invitrogen; A11012/A11005
Wheat Germ agglutinin (WGA) Alexa Fluor 488	1:100	CellSignalling Tech; 13116S

Supplemental Table S2: Density of Meso-ECTs input versus compacted

Input Density (hiPSC-CM/mL)	Calculated Volume (D7, mm^3)	Density after Compaction (hiPSC-CM/mL)
5M/mL	0.55±0.06	318.18M±64.,28M
15M/mL	1.045±0.06	502.39M±53.03M
30M/mL	1.98±0.11	530.3M±59.12M
50M/mL	3.91±0.28	448.14M±63.43M

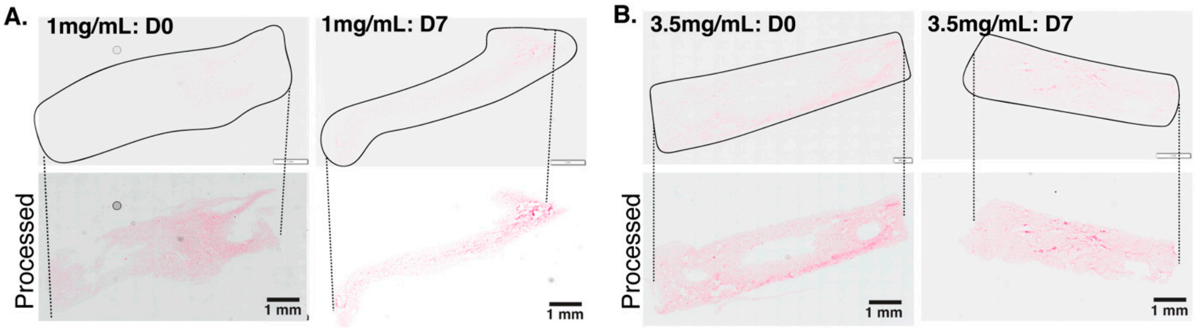

Supplemental Figure S1: Representative histological staining of Picrosirius Red/Fast Green (PRFG) for acellular 1mg/mL collagen constructs. (A) Day 0 (D0) and **(B)** Day 7 (D7) of culture of acellular constructs (top row). Heavy processing was used (bottom row) in order to visualize acellular constructs boundaries.

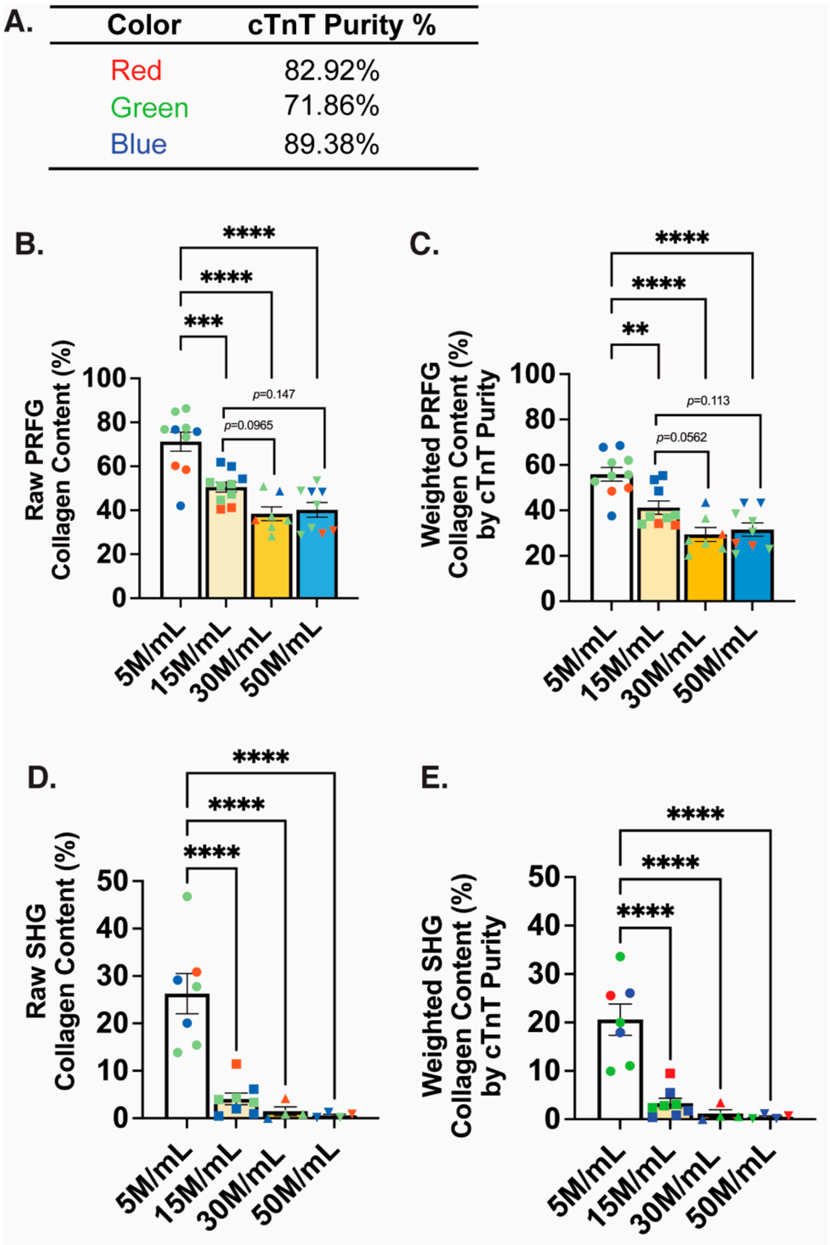

Supplemental Figure S2: Meso-ECT collagen content quantified by Picrosirius Red/Fast Green (PRFG) and Second Harmonic Generation (SHG) imaging normalized by cardiac differentiation batch purity. (A) Purity of cardiac differentiation batches as assessed by cTnT+ populations in flow cytometry. **(B-C)** Collagen content quantified by PRFG assessing raw values and values normalized by cardiac differentiation purity. **(D-E)** Similarly, for SHG analysis with collagen content quantification assessed by raw values and normalized by cardiac differentiation purity. n = 7-10 analyzed tissues per group for PRFG staining and n = 4-7 for SHG with multiple areas averaged to analyze per tissue; ***p*<0.01; ****p*<0.001, *****p*<0.0001.

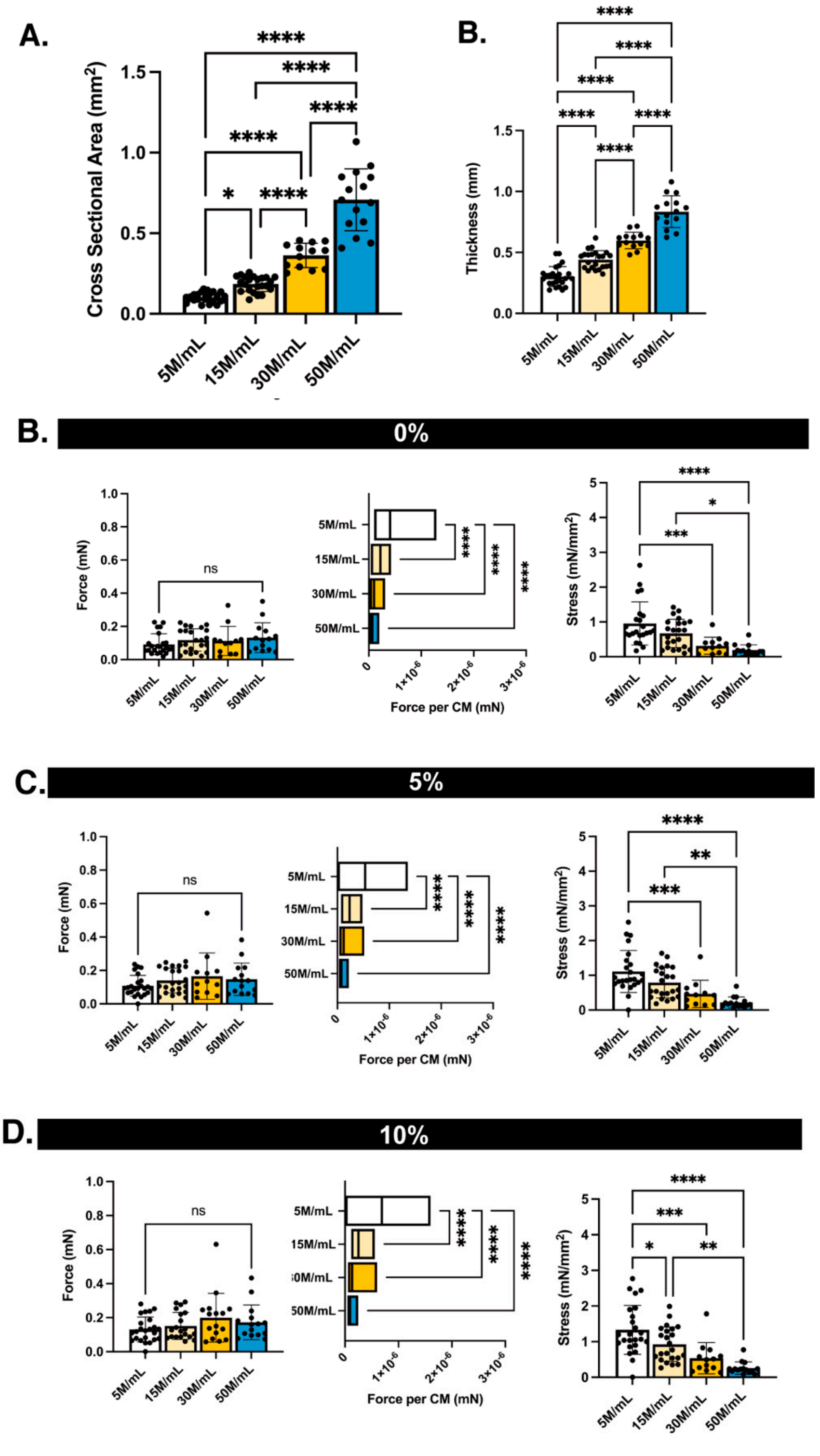
A.
Cross Sectional Area (mm2)
5M/mL
15M/mL
30M/mL
50M/mL
B.
Thickness (mm)
B.
0%
Force (mN)
ns
Force per CM (mN)
Stress (mN/mm2)
C.
5%
Force (mN)
ns
Force per CM (mN)
Stress (mN/mm2)
D.
10%
Force (mN)
ns
Force per CM (mN)
Stress (mN/mm2)

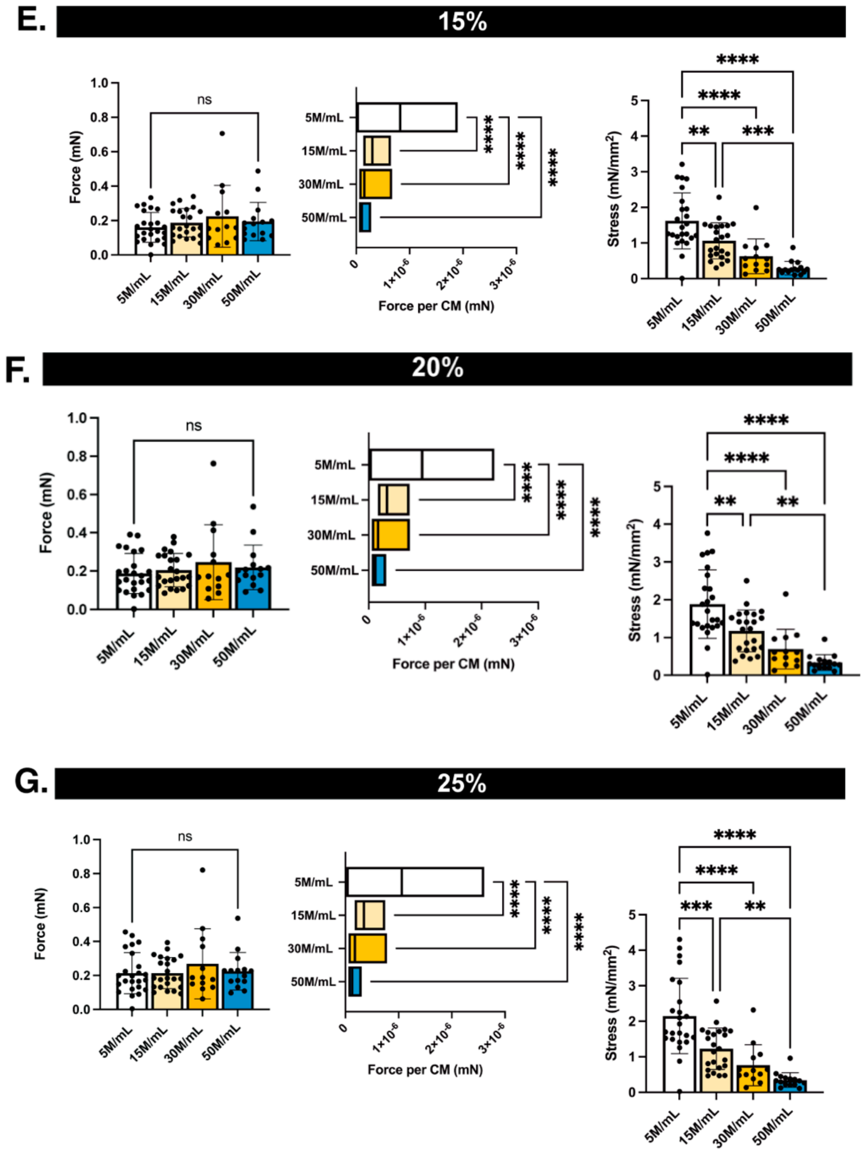

Supplemental Figure S3: In depth active mechanical analysis of contractile magnitude for meso-ECTs. (A) Cross-sectional area (CSA) of ECTs calculated as an ellipse from measured tissue width and thickness used in the normalization of contractile force to calculate stress. **(B-G)** Force generation (mN), Force normalized by input of hiPSC-CM (mN/number of hiPSC-CMs) and Stress (mN/mm^2) for 0-25% stretch under 1Hz pacing. n = 15-24 tissues analyzed per condition; *p<0.5; **p<0.01; ***p<0.001, ****p<0.0001.

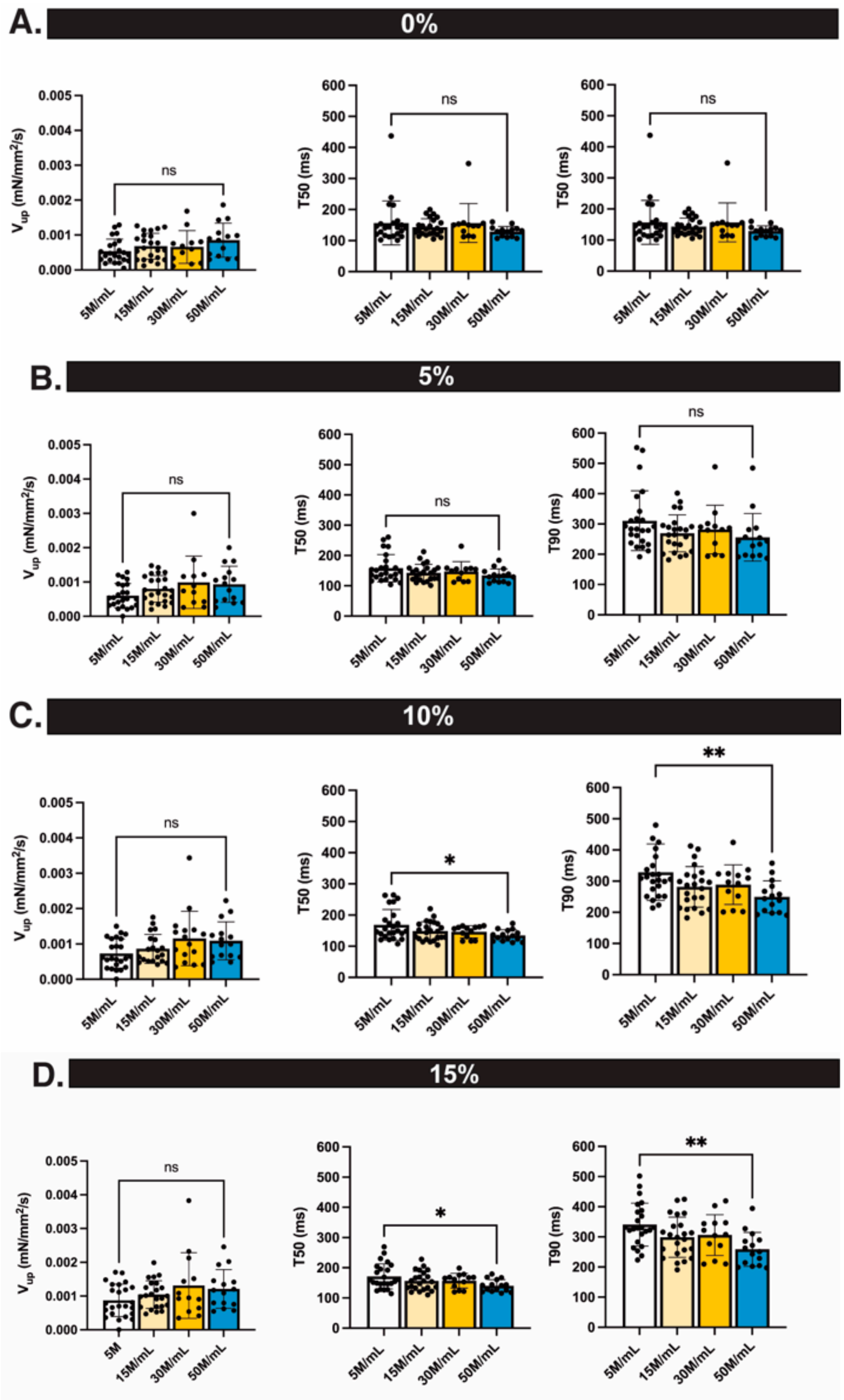

A.
0%
V_{up} (mN/mm²/s)
T50 (ms)
T50 (ms)
ns
5M/mL
15M/mL
30M/mL
50M/mL
B.
5%
V_{up} (mN/mm²/s)
T50 (ms)
T90 (ms)
ns
C.
10%
V_{up} (mN/mm²/s)
T50 (ms)
T90 (ms)
ns
*
**
D.
15%
V_{up} (mN/mm²/s)
T50 (ms)
T90 (ms)
5M
ns
*
**

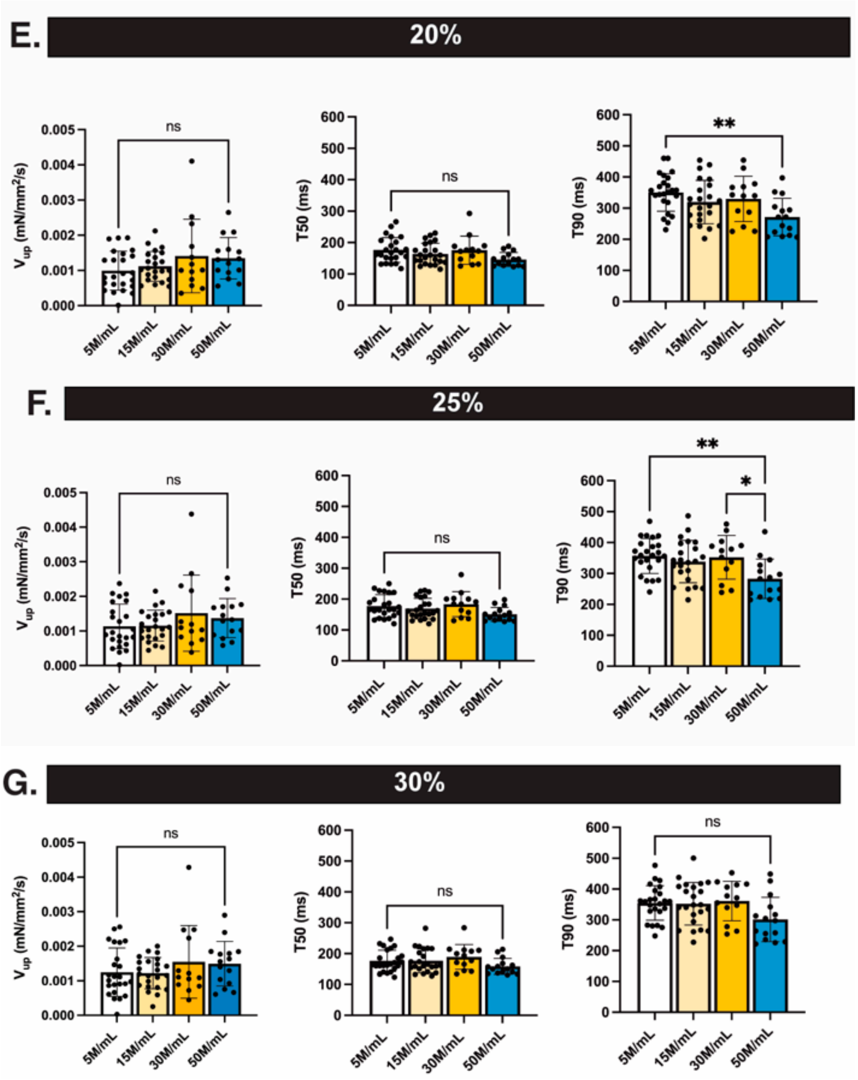

Supplemental Figure S4: In depth active mechanical analysis of contractile kinetics for meso-ECTs. (A-G) Upstroke Velocity (V_{up}, mN/mm^2/s), Time to 50% relaxation (T50, ms) and Time to 90% relaxation (T90, ms) from 0-30% stretch under 1Hz pacing. n = 15-24 tissues analyzed per condition; *p<0.5; **p<0.01; ***p<0.001, ****p<0.0001.

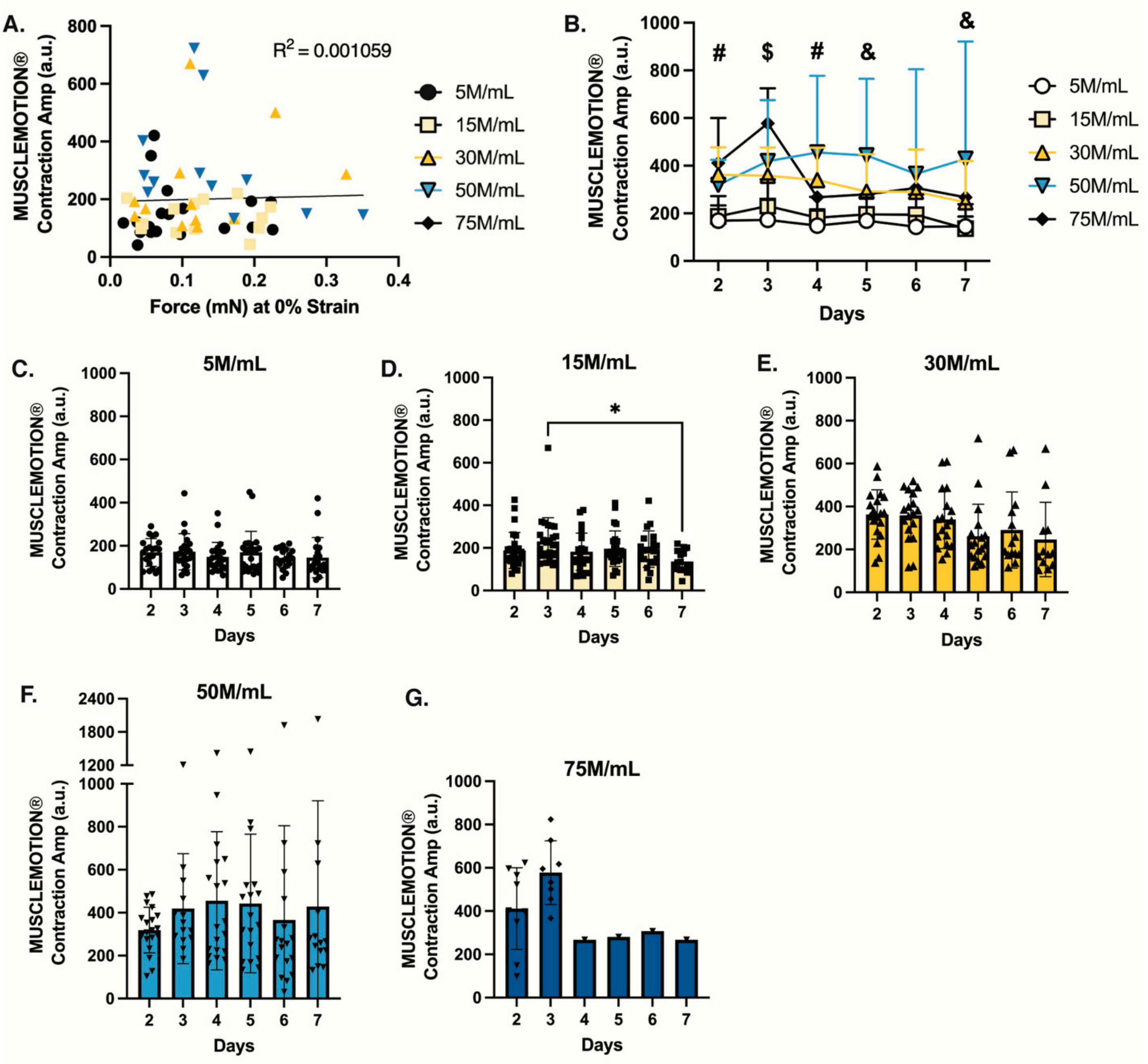

Supplemental Figure S5: Video-based analysis of active mechanics of meso-ECTs using MUSCLEMOTION® software. (A) Correlation of contraction amplitude quantified using MUSCLEMOTION® analysis of videos taken of mezzo-ECTs at Day 7 in culture and force measurements acquired from uniaxial tensile testing at 0% stretch. **(B)** Serial measurements of contraction amplitude for each density condition throughout the 7-day in vitro culture. **(C-G)** Contraction amplitude plotted as a bar graph for each density condition 5M/mL, 15M/mL, 30M/mL, 50M/mL and 75M/mL over the 7-day in vitro culture. n = 9-24 tissues analyzed per condition; # indicates all conditions are significant to 5M/mL and 15M/mL except the comparison between 5M/mL and 15M/mL with a minimum $p<0.05$; $ indicates the significance of # with added significant comparison between 30M/mL and 75M/mL with a minimum $p<0.05$; & indicates the significance of 5M/mL and 15M/mL compared to 50M/mL with a minimum $p<0.05$.

A.

C.

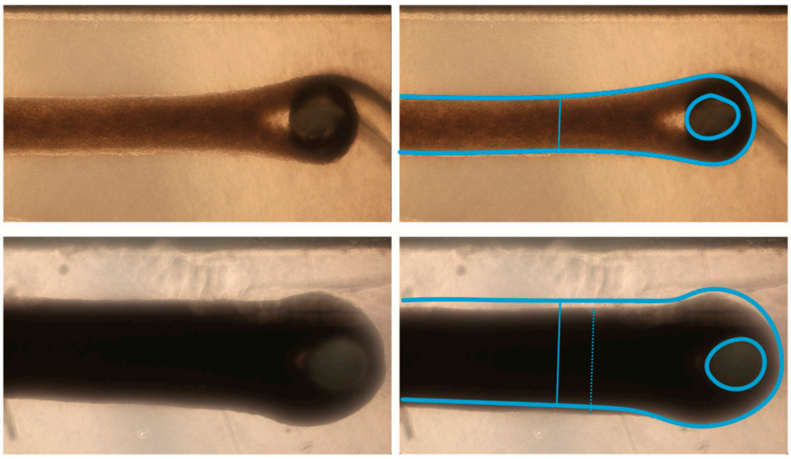

B.

5M/mL

Mechanics	Readout
Uniaxial Force at 0% Strain	0.158 mN
MUSCLEMOTION contraction amplitude	99.360 a.u.

D.

50M/mL

Mechanics	Readout
Uniaxial Force at 0% Strain	0.085nmN
MUSCLEMOTION contraction amplitude	2030.525 a.u.

Supplemental Figure S6: MUSCLEMOTION® analysis of meso-ECTs. (A) Frames from 5M/mL meso-tissue video of contraction. **(B)** Quantification of 5M/mL meso-ECT contraction measured by uniaxial force at 0% strain and MUSCLEMOTION® analysis with video taken on Day 7. **(C)** Frames from 50M/mL meso-tissue video of contraction. **(D)** Quantification of 50M/mL meso-ECT contraction measured by uniaxial force at 0% strain and MUSCLEMOTION® analysis with video taken on Day 7.

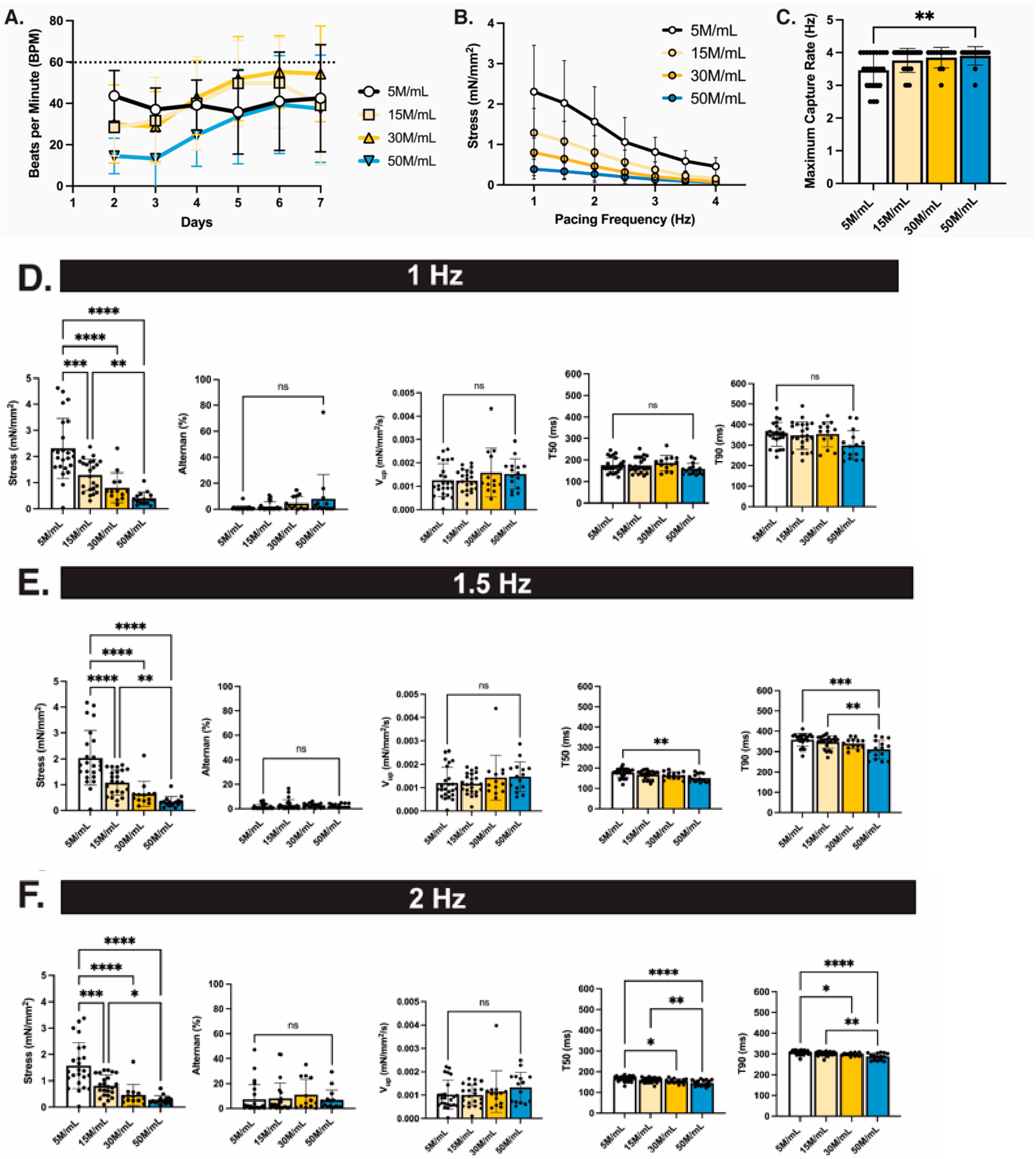
A.
Beats per Minute (BPM)
Days
5M/mL
15M/mL
30M/mL
50M/mL
B.
Stress (mN/mm²)
Pacing Frequency (Hz)
5M/mL
15M/mL
30M/mL
50M/mL
C.
Maximum Capture Rate (Hz)
**
5M/mL
15M/mL
30M/mL
50M/mL
D.
1 Hz
Stress (mN/mm²)
Alternan (%)
Vup (mN/mm²/s)
T50 (ms)
T90 (ms)
****
***
**
ns
E.
1.5 Hz
Stress (mN/mm²)
Alternan (%)
Vup (mN/mm²/s)
T50 (ms)
T90 (ms)
****
**
***
ns
F.
2 Hz
Stress (mN/mm²)
Alternan (%)
Vup (mN/mm²/s)
T50 (ms)
T90 (ms)
****
***
**
*
ns

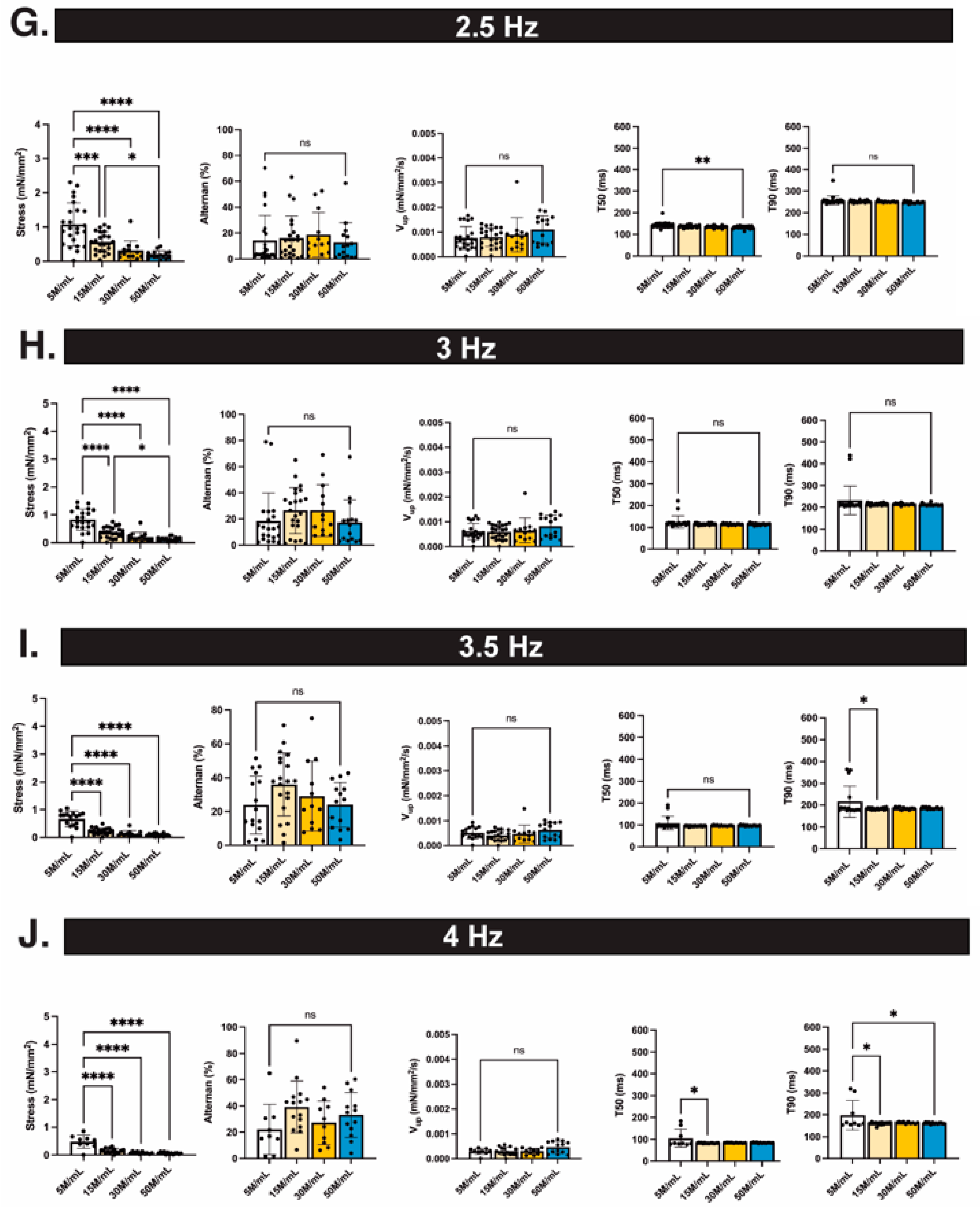

Supplemental Figure S7: Stress-frequency response of contractile magnitude and kinetics for meso-ECTs. (A) Beats per minute (BPM) of mezzo-ECTs while in culture under 1Hz stimulation. **(B)** Stress-frequency response of mezzo-ECTs from 1Hz-4Hz stimulation at 30% stretch during mechanical uniaxial testing. **(C)** Maximum capture rate (MCR, Hz) achieved for each mezzo-ECT density condition during force-frequency testing. **(D-J)** Stress generation (mN/mm^2), Alternans (%), Upstroke Velocity (V_{up}, $mN/mm^2/s$), Time to 50% relaxation (T50, ms) and Time to 90% relaxation (T90, ms) from 1-4Hz pacing. n = 15-24 tissues analyzed per condition; **p*<0.5; ***p*<0.01; ****p*<0.001, *****p*<0.0001.

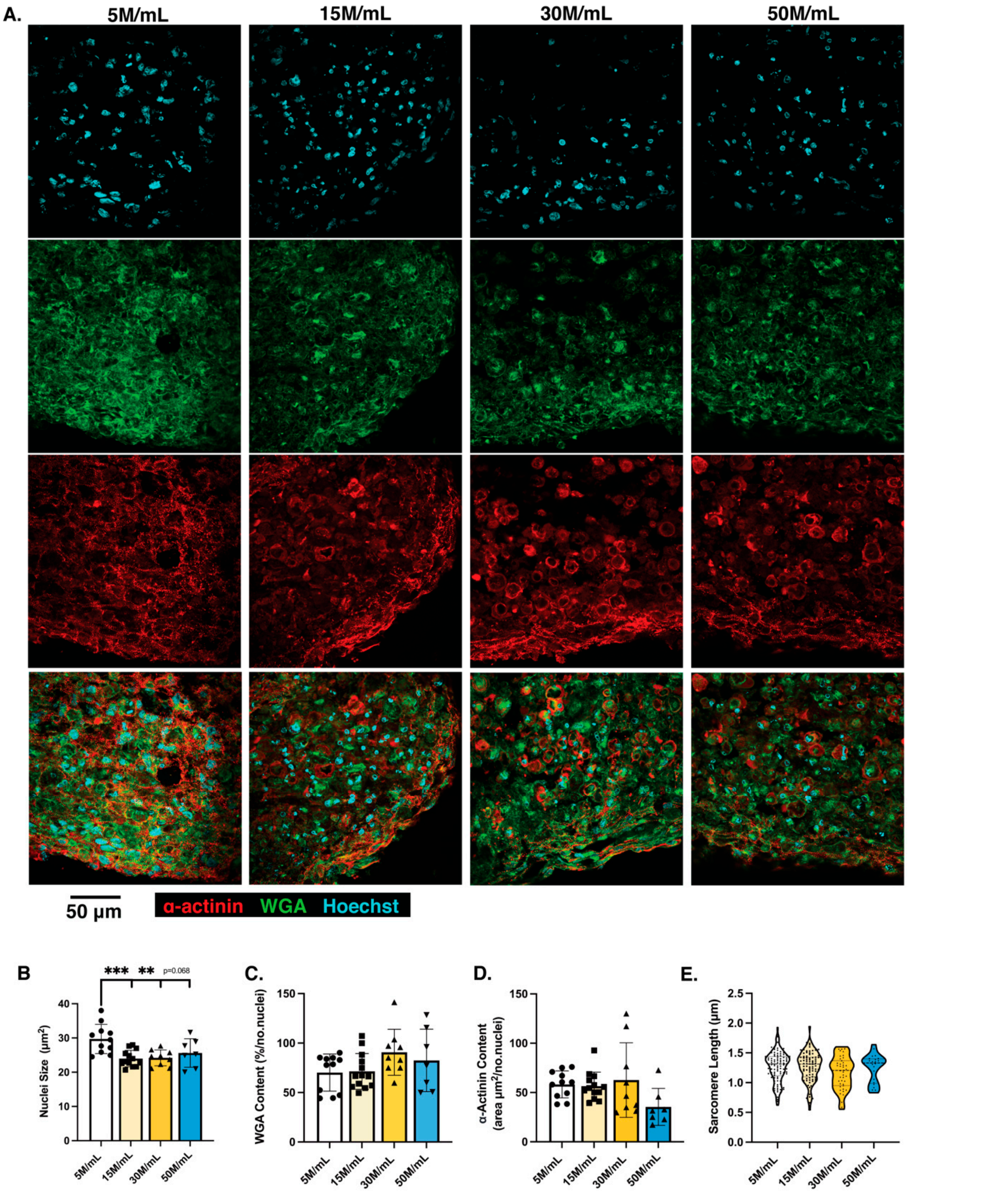

Supplemental Figure S8: Meso-ECTs histological staining and quantification using Hoechst (nuclear marker), WGA (cell surface) and α-actinin (sarcomere marker). (A) Quantification of nuclear size, as measured by area. **(B)** Quantification of WGA content normalized by the number of nuclei; **(C)** Quantification of α-actinin content normalized by the number of nuclei; **(D)** Quantification of sarcomere length and relative prevalence of aligned sarcomeres. $^{**}p<0.01$; $^{***}p<0.001$.

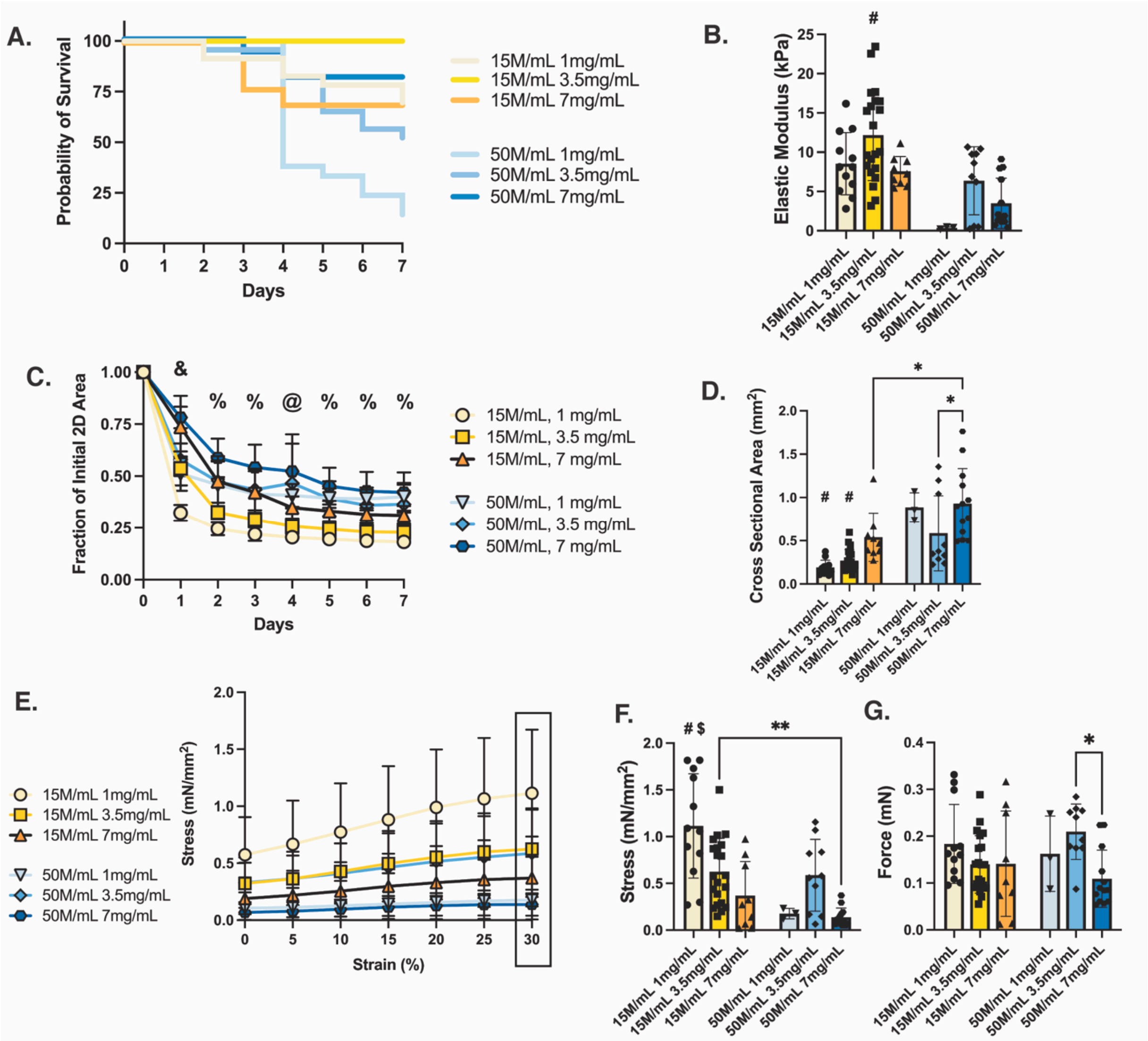

Supplemental Figure S9: Increasing collagen content in meso-ECTs. (A) Survival curve showing percentage of intact meso-ECTs to assess structural survival; **(B)** Elastic modulus of tissues to assess passive mechanical properties. **(C)** Quantification of tissue compaction over 7-day culture. **(E)** Active stress generation of meso-ECTs from 0-30% stretch, measured at increments of 5%. **(F)** Stress and **(G)** Force within the meso-ECTs at 30% stretch. n = 13-23 samples per group with significance defined as **p*<0.5; ***p*<0.01. & indicates all comparisons except 15M/ml 3.5mg/mL to all 50M/mL conditions are significant to each other at a minimum *p*<0.05; % indicates all comparisons except 50M/mL 1mg/mL vs 3.5 mg/mL are significant to each other at a minimum *p*<0.05; @ indicates all comparisons except 15M/mL 1mg/mL vs 3.5 mg/mL and 50M/mL 1mg/mL vs 3.5 mg/mL are significant to each other at a minimum *p*<0.05; # indicates all comparisons to the different density are significant to each other at a minimum *p*<0.05; $ indicates all conditions within the same are significant to each other at a minimum *p*<0.05; # indicates all conditions except the comparison between 5M/mL and 15M/mL are significant with a minimum *p*<0.05.

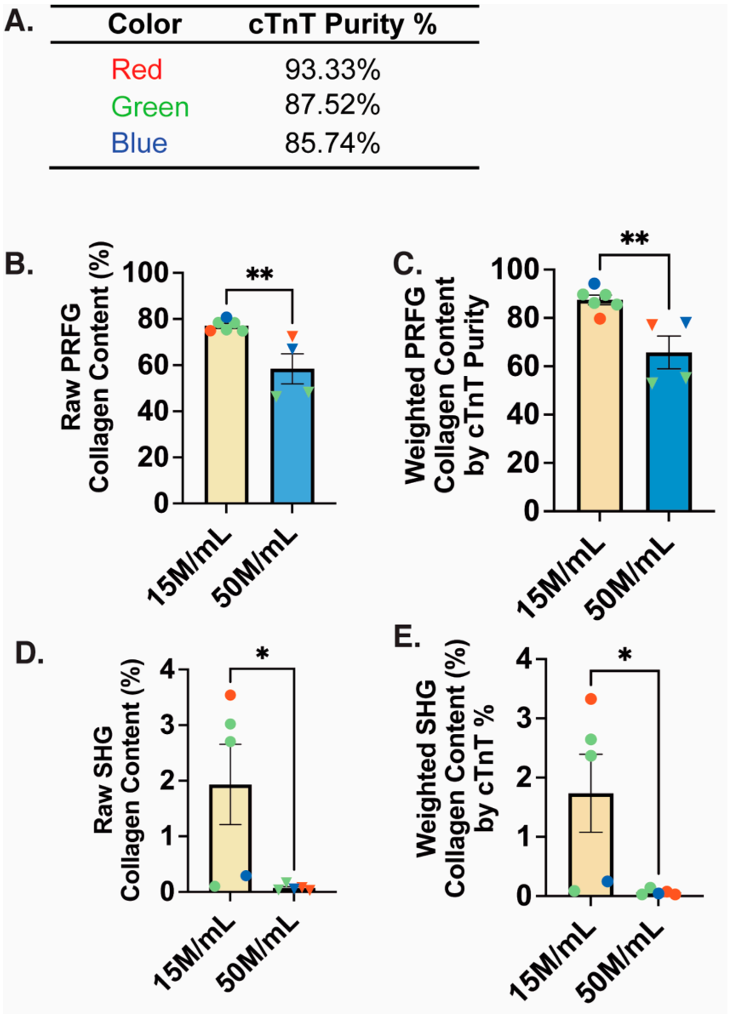

Supplemental Figure S10. Macro-ECT collagen content quantified by Picrosirius Red/Fast Green (PRFG) and Second Harmonic Imaging normalized by cardiac differentiation batch purity. (A) Purity of cardiac differentiation batches as assessed by cTnT+ populations in flow cytometry. **(B-C)** Collagen content quantified by PRFG assessing raw values and values normalized by cardiac differentiation purity. **(D-E)** Similarly, for SHG analysis with collagen content quantification assessed by raw values and normalized by cardiac differentiation purity. *p<0.5; **p<0.01.

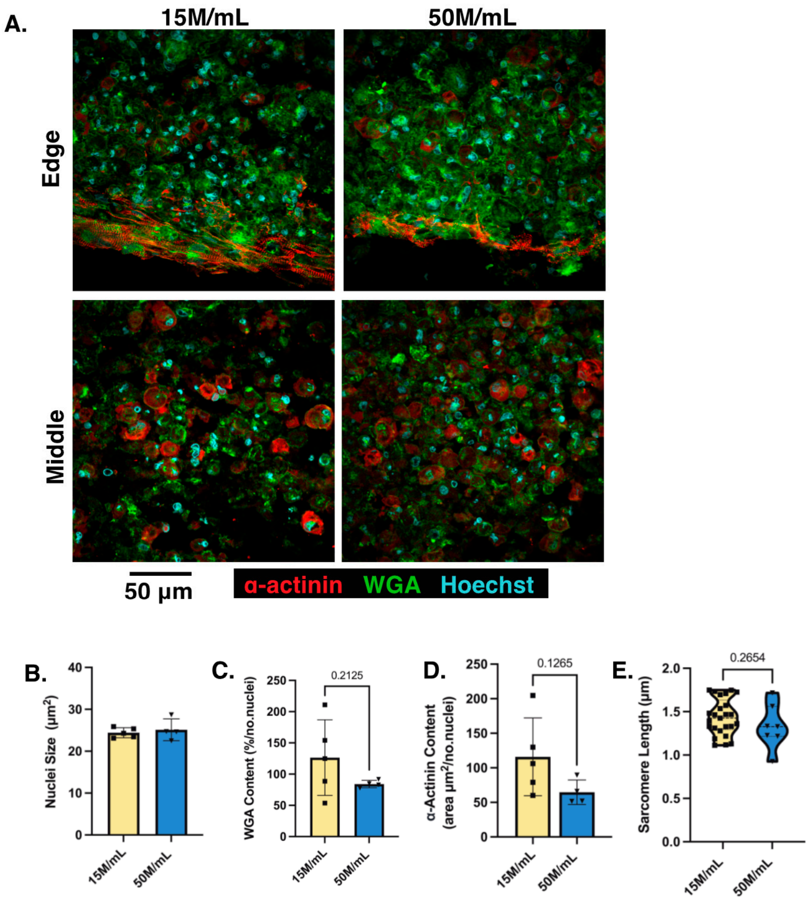

Supplemental Figure S11. Quantification of macro-ECTs histological staining using Hoechst (nuclear marker), WGA (cell surface) and α-actinin (sarcomere marker). (A) Immunohistochemical staining of macro-ECTs at the tissue edge and middle. **(B)** Quantification of nuclear size, as measured by area. **(C)** Quantification of WGA content normalized by the number of nuclei; **(D)** Quantification of α-actinin content normalized by the number of nuclei; **(E)** Quantification of sarcomere length and relative prevalence of aligned sarcomeres.

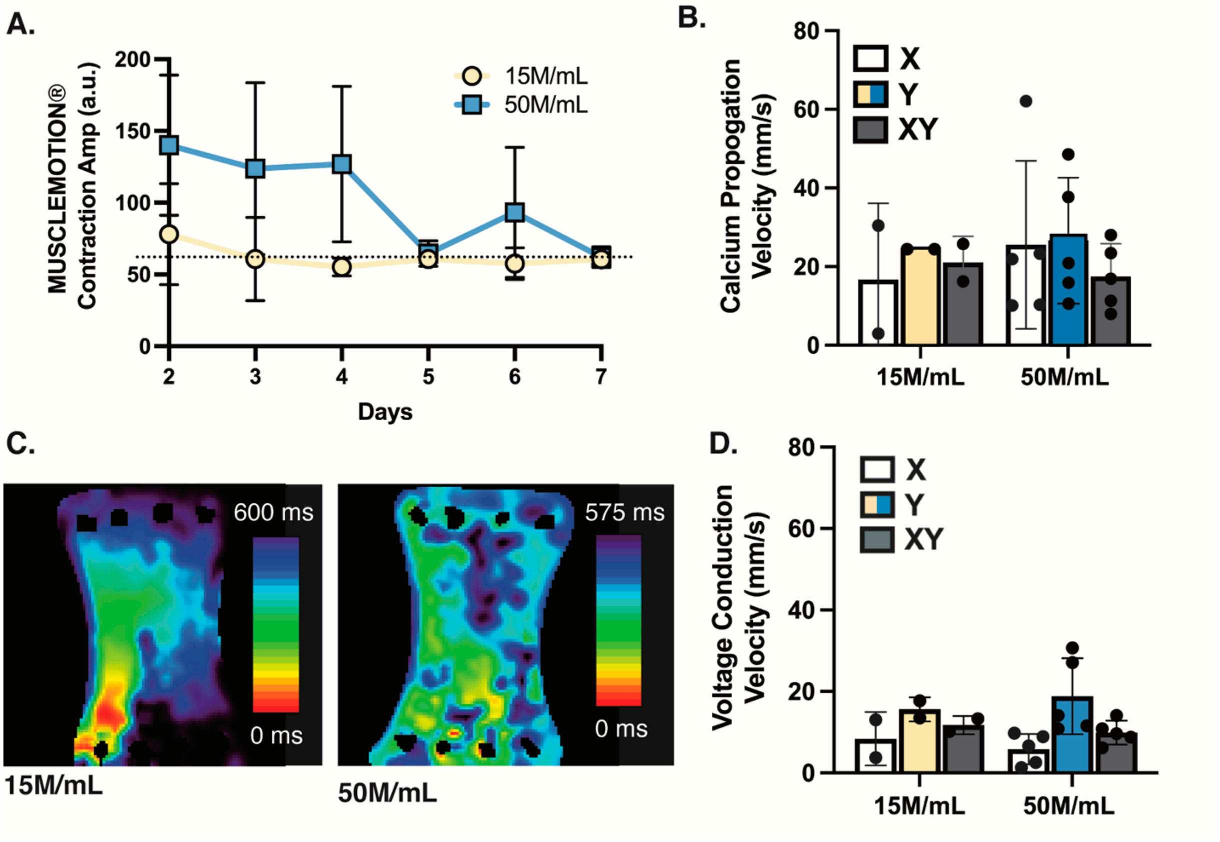

Supplemental Figure S12. Optical mapping of macro-ECTs. (A) Beats per minute (BPM) of macro-ECTs while in culture under 1Hz stimulation. **(B)** Quantification of calcium propagation velocity; **(C)** Heatmap of voltage propagation for 15M/mL and 50M/mL macro-ECTs; **(D)** Quantification of voltage conduction velocity

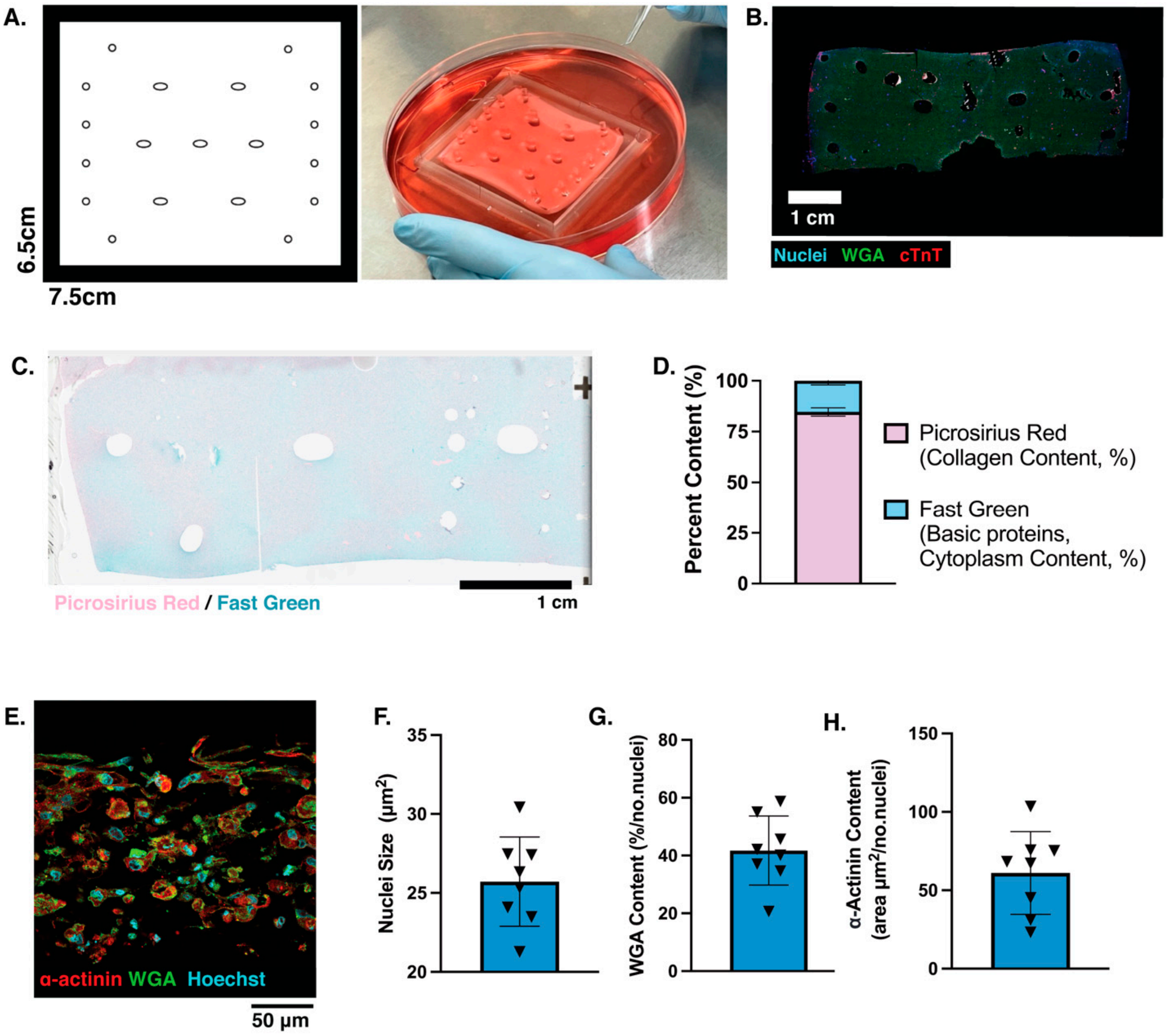

Supplemental Figure S13. Mega-ECT fabrication and structural analysis. (A) Mold and culture system utilized to fabricate the mega-ECT. **(B)** Half-scale histology image of mega-ECT (longitudinal section). **(C)** Half-scale PRFG stain of mega-ECT (longitudinal section); **(D)** Quantification of PRFG stain; **(E)** Representative histology staining of α-sarcomeric actinin (α-actinin), wheat germ agglutin (WGA) and Hoechst (longitudinal section); **(F)** Quantification of nuclear size, as measured by area; **(G)** Quantification of WGA content normalized by the number of nuclei; **(H)** Quantification of α-actinin content normalized by the number of nuclei. N = 2 implanted in swine model; n = 1 analyzed for histology with multiple regions averaged for all quantification.

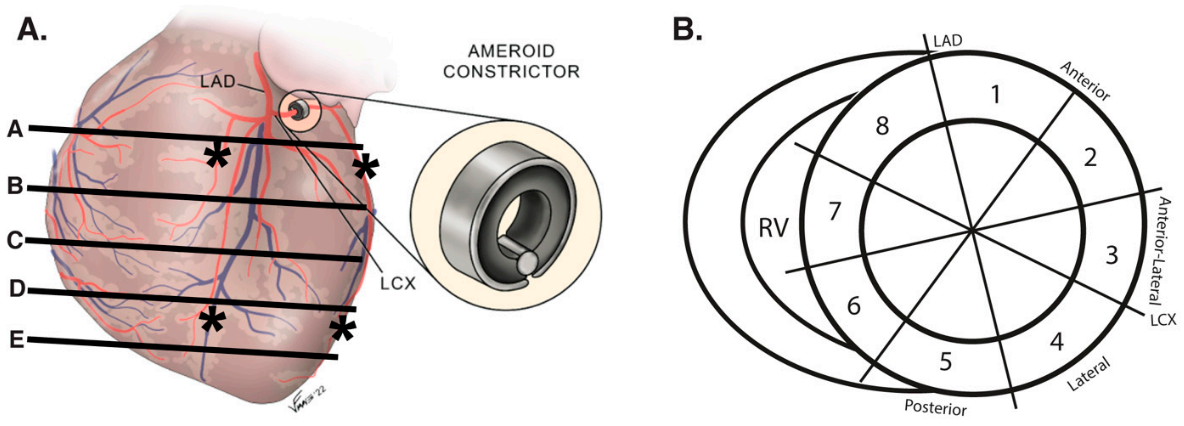

Supplemental Figure S14. Locations of colorimetric and immunohistochemical staining performed after explant of swine heart implanted with mega-ECT. (A) Left ventricle (LV) view with lines A-E representing locations where colorimetric staining was performed to determine scar morphology; asterisk represent the approximate corners of the implanted mega-ECT. **(B)** Cross-section of the heart with nomenclature numbers for identification of subsampled regions where immunohistochemical staining was performed to identify engrafted hiPSC-CMs.

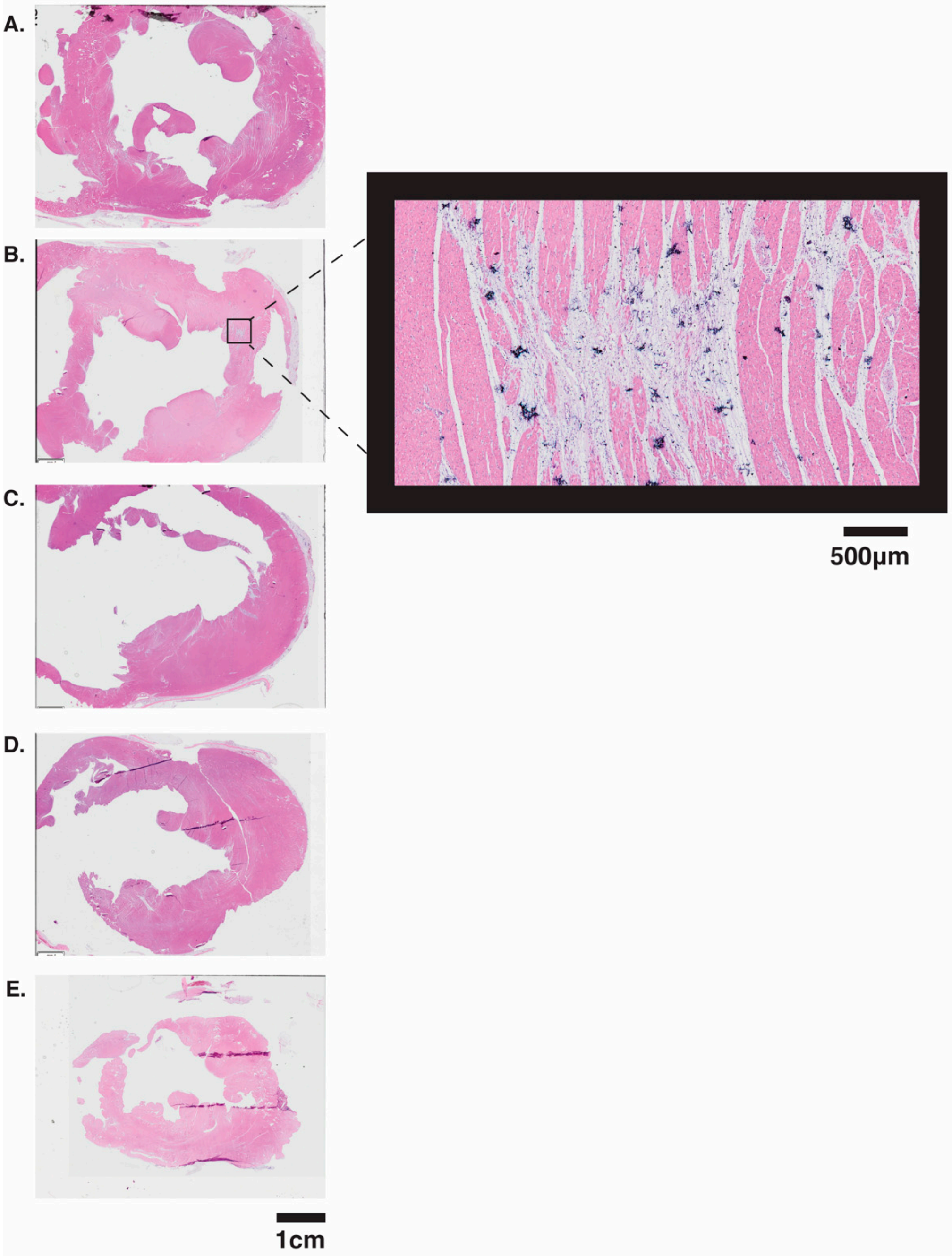

Supplemental Figure S15. Hematoxylin and Eosin (H&E) staining of explanted swine heart. (A-E) Sections from base to apex.

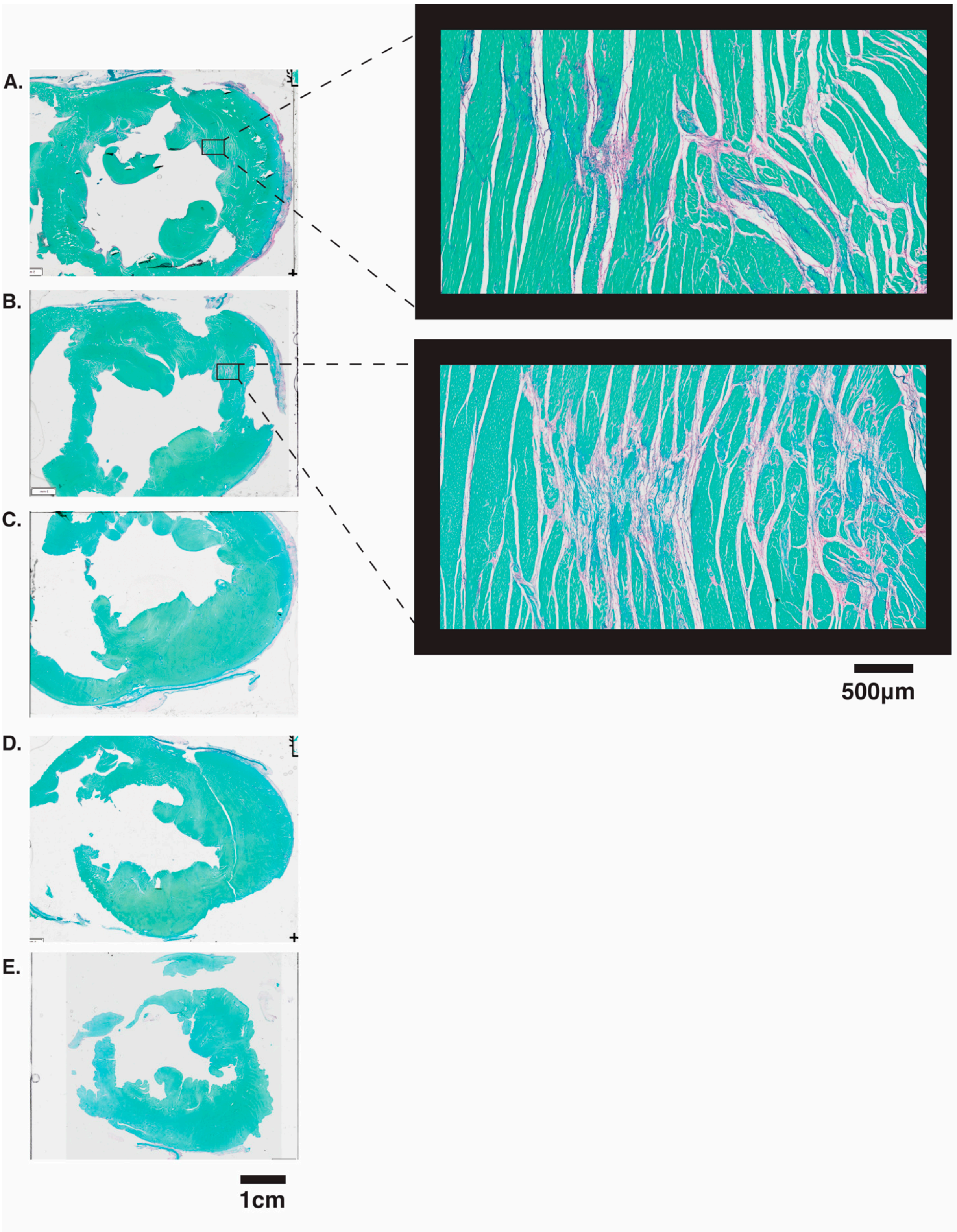

Supplemental Figure S16. Picrosirius Red/ Fast Green staining of explanted swine heart. (A-E) Sections from base to apex.

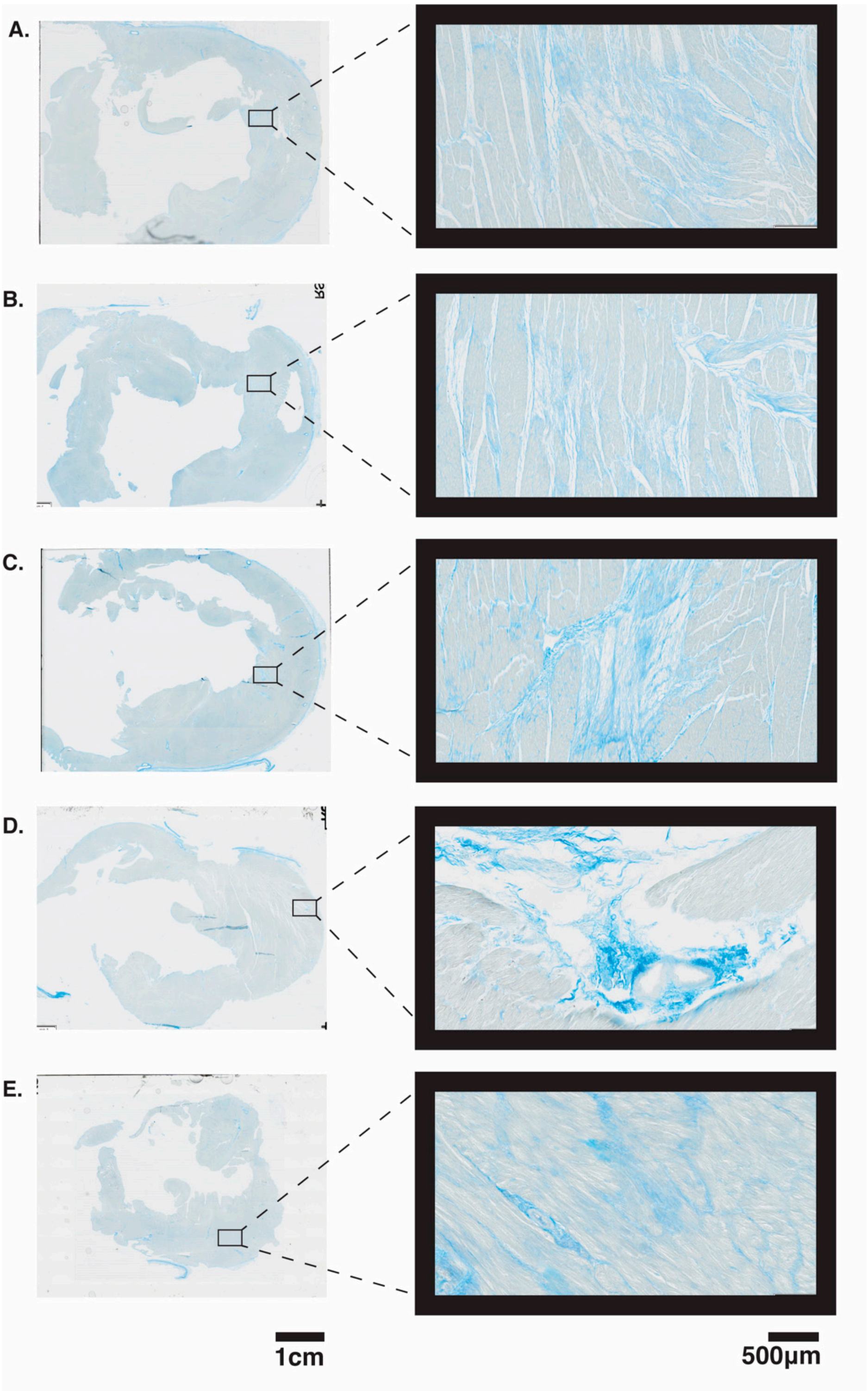

Supplemental Figure S17. Aniline blue collagen staining of explanted swine heart. (A-E) Sections from base to apex.

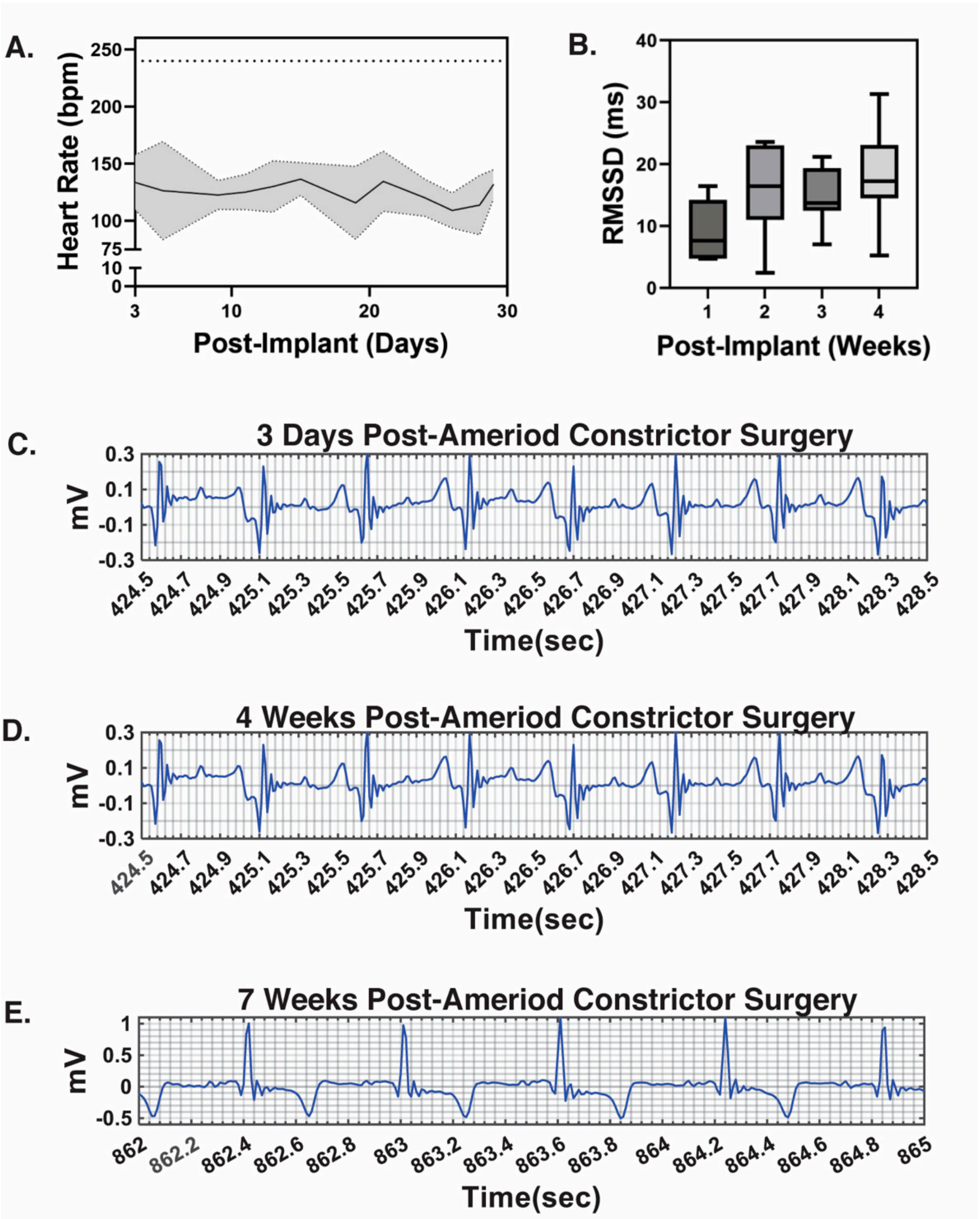

Supplemental Figure S18. Electrophysiology analysis of swine model throughout study. (A) Heart rate measured in beats per minute (BPM) of swine throughout the study. **(B)** Quantification of the root mean square of successive differences between normal heartbeats (RMSSD). **(C)** ECG graph 3-days post ameroid constrictor surgery. **(D)** ECG graph 4-weeks post ameroid constrictor surgery. **(E)** ECG graph 7-weeks post ameroid constrictor surgery.

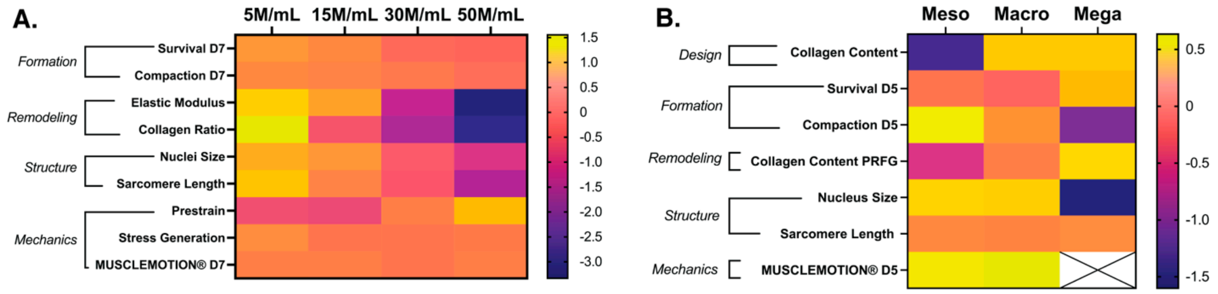

Supplemental Figure S19. Direct comparison of quantified metrics for cell density and ECT size. For both heatmaps, the fraction of the average value of that metric across groups was taken and log_2 transformed. **(A)** Heatmap of different hiPSC-CM dose within the meso-ECT format. Survival average (75.2%), Compaction average (0.73, calculated as 1- fraction of initial area), Collagen content ratio SHG to PRFG average (12.58% area), Prestrain average (19.70%), Elastic Modulus average (6.73 kPa), Stress generation average (1.20 mN/mm^2), Nuclei size average (25.89 µm^2), Sarcomere length average (1.24 µm). **(B)** Heatmap of constant 50M/mL density with different ECT size. Collagen hydrogel concentration average (2.67 mg/mL), Survival at D5 average (80.24%), Compaction at D5 average (0.42, calculated as 1- fraction of initial area), Collagen content PRFG average (61.10%), MUSCLEMOTION® contraction amplitude D5 average (329.73 a.u.), Nuclei size average (25.48 µm^2), Sarcomere length average (0.856 µm). Please note PRFG collagen content and sarcomere length were performed on D7 for meso- and macro-ECTs and D5 for mega-ECT; the X indicates a fraction value of 0.
